# Constructing a digital twin maturity assessment framework for the building construction phase based on an improved matter-element model: A case study of a construction project in Xinyang, China

**DOI:** 10.1371/journal.pone.0332449

**Published:** 2025-09-29

**Authors:** Qi Yang, Zongjun Xia, Xiaodan Li, Zhen Liu, Zhifei Chen, Jing Li, Yangyang Wang, Jinfeng Wang

**Affiliations:** 1 State Key Laboratory for Tunnel Engineering, Beijing, China; 2 China University of Mining and Technology, Beijing, China; 3 China RAILWAY 15 BUREAU Group Corporation, Shanghai, China; Jimma University College of Agriculture and Veterinary Medicine, ETHIOPIA

## Abstract

Digital twin technology has the potential to enhance construction efficiency, reduce costs, and minimize errors. However, its application during the construction phase remains at an early stage, largely constrained by the absence of standardized guidelines and principles. To address this challenge, it is essential to establish a comprehensive and universal maturity assessment framework to facilitate the effective implementation of this technology in the construction phase of building projects. This study focuses on two critical aspects: the development of the maturity assessment framework and its empirical validation. The proposed framework encompasses a maturity assessment indicator system covering five dimensions: acquisition layer, data layer, modeling layer, analysis layer, and application layer. For the first time, an optimized matter-element model based on dynamic thresholds and nonlinear correlation is introduced to improve the accuracy of maturity assessments. Furthermore, a feedback mechanism based on Importance-Performance Analysis (IPA) is utilized to clarify the formulation of optimization strategies. Finally, the framework is applied to the CAZ Innovation Industrial Park construction phase in Xinyang, Henan Province. The assessment results demonstrate that the system precisely measures the project’s maturity level and provides effective improvement recommendations. This study not only offers technological support for assessing and optimizing the digital twin maturity during the construction phase of building projects but also provides methodological insights into global digital twin maturity assessments.

## 1. Introduction

Digital Twin (DT) technology refers to the virtual representation of physical assets, processes, or systems. Through real-time monitoring and data analytics, DT enables performance prediction and optimization [1]. This innovative approach has been shown to significantly enhance energy efficiency [2], thereby contributing to the sustainable development of the construction industry. Since the early 21st century, the global construction sector has progressively adopted a range of international standards and methodologies in its transition toward digitalization and intelligent systems, laying a foundational framework for the widespread implementation of DT technologies. The institutional adoption of DT in construction has evolved through a coordinated progression from methodology to principles, standards, and policy. The Capability Maturity Model Integration (CMMI), introduced in 2002 and updated in 2018, provides a cross-sectoral paradigm for process improvement and maturity assessment [1]. In 2018, the United Kingdom introduced the Gemini Principles, which established value orientation and data governance guidelines for DT applications [2]. That same year, ISO 19650−1/-2 was published, setting international standards for lifecycle information management within Building Information Modeling (BIM) environments [3]. Building upon these foundations, policy diffusion has accelerated since 2020. The European Union has promoted the comprehensive adoption of BIM among member states to enhance efficiency and sustainability (EU BIM Policy). In 2021, the United Kingdom released its Digital Twin Strategy (UK DT Strategy), while China’s Ministry of Industry and Information Technology advocated for the development of DT platforms in its 14th Five-Year Plan for Intelligent Manufacturing (MIIT Policy). In 2022, the American National Standards Institute (ANSI) introduced DT standards encompassing data exchange, model management, and cybersecurity (ANSI DT Standards). Collectively, these frameworks are mutually reinforcing: CMMI provides organizational capability and evaluation pathways; the Gemini Principles ensure alignment with public value and data governance; and ISO 19650 guarantees cross-phase coordination and information consistency. Together, they facilitate national policy implementation and support industry-wide scalability. As global urbanization accelerates, the application of DT technology in the construction sector has emerged as an inevitable trend. By offering more precise and comprehensive insights into building performance, DT has the potential to fundamentally transform the industry and contribute to the development of intelligent, resilient, and sustainable cities [3].

Digital Twin (DT) technology refers to the virtual representation of physical assets, processes, or systems. By leveraging real-time monitoring and data analysis, it enables predictions and optimization of performance [4]. This innovative technology significantly enhances energy efficiency [5], thereby fostering sustainable development in the construction industry. In recent years, the global construction sector has increasingly embraced the applications of Digital Twin technology. For instance: In 2020, the European Union released the EU Building Information Modeling (BIM) Directive (https://single-market-economy.ec.europa.eu/sectors/construction-and-real-estate/bim_en), mandating all member states to adopt BIM standards by 2020 to improve project efficiency and sustainability. In 2021, the UK government published the UK Digital Twin Building Strategy (https://www.gov.uk/government/publications/digital-twin-strategy), which highlights the critical role of Digital Twin technology in enhancing the efficiency and sustainability of the construction sector and outlines various measures to promote its adoption.In the same year, China’s Ministry of Industry and Information Technology released the “14th Five-Year Plan for Intelligent Manufacturing Development” (https://www.miit.gov.cn/), advocating for the establishment of Digital Twin platforms, particularly in smart city applications. In 2022, the American National Standards Institute introduced the ANSI Digital Twin Building Standards (https://www.ansi.org/), which address aspects such as data exchange, model management, and security, aiming to promote widespread application of the technology in the U.S. construction industry. As urbanization continues to accelerate globally, the application of Digital Twin technology in the construction domain has become increasingly indispensable. This technology provides more accurate and comprehensive information about building performance, potentially revolutionizing the construction industry and contributing to the development of intelligent, resilient, and sustainable cities [6].

Digital Twin (DT) technology was initially applied in the aerospace field [7,8]. With the development of new technologies such as the Industrial Internet, Internet of Things (IoT), cloud computing, big data, and artificial intelligence, digital twin applications have expanded into a wide range of industries [9]. In the construction industry, research on DT primarily focuses on applications during the project design, construction, and operation and maintenance phases [10–12]. Among these, the construction phase constitutes a critical part of the building lifecycle [13]. The application of digital twin technology during this phase helps improve construction efficiency and quality while reducing costs and minimizing errors [5]. At present, DT technology is mainly used during the construction phase to assist in various management activities, such as construction safety management [14–17], uncertain site scheduling [18], fault detection, and quality management [19]. Related research topics can be broadly categorized into three areas: **(1) Exploration of DT Implementation Frameworks:** For instance, Boje C et al. proposed a conceptual framework for constructing digital twins from the perspectives of service, value chains, and methods [20]. Similarly, Pan Y developed a digital twin framework for structural health monitoring based on cloud computing and deep learning [21]; **(2) Analysis of Specific DT Applications in Construction Processes:** For example, Kaewunruen S and N. Xu explored the specific application of BIM technology in railway station buildings from a digital twin perspective, finding that hollow wall insulation combined with fire barrier installation achieved the optimal carbon emission rate among all tested schemes [22]. Gerhard D et al. proposed a fault-tolerant and rapid module production method, using prefabricated, freely extensible high-performance concrete components conceptualized within a digital twin framework [23]. Additionally, Angjeliu G developed a simulation model for DT applications in historic masonry buildings to analyze structural behavior during different construction phases [24]; **(3) Optimization of DT Technology in Construction:** For instance, Chen S et al. proposed a novel method based on multi-view consistency constraints and deep learning to refine the missing regions in depth maps generated by MVS algorithms, providing improved technical support for DT systems [25]. Ritto T G et al. introduced a digital twin conceptual framework for dynamic structural damage issues, combining physical models with machine learning to enhance the accuracy of digital twins for damaged structures [26].

Digital Twin Maturity is utilized to evaluate the developmental level of digital twin systems, playing a crucial role in enabling enterprises to assess their digital twin application level and formulate future improvement strategies [27]. Scholars from various disciplines have conducted research on digital twin maturity assessment. **The evaluation model frameworks primarily include:** (1) “Digital Model-Digital Shadow-Digital Twin” Framework based on Information Flow [28]; (2)“Digital Twin Capability-Phase Objectives-Technical Requirements” Model based on Development Levels [29]; (3) “Purpose-Trust-Function” Model based on Asset Management [30]; (4) “Context-Data-Computing Capacity-Model-Integration-Control-Human-Machine Interface” Framework based on Lifecycle [31]; (5) “Value-Function-Reliability” Model Framework based on Equipment Attributes [32]. **The evaluation methods primarily include:** (1) Analytic Hierarchy Process (AHP) [33]; (2) Decision-Making Trial and Evaluation Laboratory (DEMATEL) Method [34]; (3) Weighted Arithmetic Mean Method [31]; (4) Probability Distribution Function Method [35]; D-ANP Method [36].

It is worth noting that significant progress has been made in evaluating the maturity of digital twins (DT) in the construction sector [34,37]. Specific advancements include:

[1] **In terms of evaluation frameworks**:1) Deng et al. [38] developed a five-level hierarchical classification system based on the building lifecycle to reflect the latest status of digital twin applications in the built environment; 2) Chen Z. S. et al. [39] proposed a maturity model comprising seven dimensions—instrument assets, models, data, interactions, functional services, systems, and organizations—to assess building digital twin projects. They emphasized that evaluation indicators should capture the temporal characteristics of the building lifecycle; 3) Afzal M. et al. [40] discussed the maturity levels of DT technologies in the construction sector based on the key components of the latest DT technologies. They proposed four distinct DT maturity stages: pre-DT, DT, adaptive DT, and intelligent DT. They further emphasized that learning capability is a key factor in enhancing DT maturity levels.
[2] **In terms of evaluation methods**: 1) Chen Z. S. et al. [39] constructed an evaluation system to assess the maturity of construction DT projects by using a multi-objective optimization model based on fairness awareness, combined with a collective opinion generation paradigm. This approach excels in consolidating information for construction DT maturity assessments involving multiple stakeholders but requires high data accuracy and adherence to specific probability distributions; 2) Chen et al. [35] developed a maturity assessment framework for Building Information Modeling (BIM) projects in the architectural design and construction phases. They employed the Large-Scale Group Decision-Making (LSGDM) method to determine maturity levels. however, this approach still retains a degree of subjectivity; 3) Alnaser A. et al. [41] employed Structural Equation Modeling (SEM) to evaluate the maturity of BIM-DT adoption in the sustainable construction domain. This innovative approach aids in understanding the complex relationships among key factors but requires a large sample size and high-quality data, and its computational process is complex.

These studies confirm the feasibility of using maturity models to assess digital twin capabilities in the construction domain. However, the development of DT maturity models remains in its infancy. Particularly during the construction phase, literature related to the evaluation of digital twin maturity is still limited. **The few existing studies include:** (1) Li T. et al. [42] proposed five maturity levels specific to the characteristics of underground infrastructure. They constructed an evaluation indicator system comprising 5 dimensions and 13 rating criteria, and employed the D-ANP method to assess maturity levels; (2) Wei Y. et al. [43] developed an off-site construction digital twin model, encompassing data structures, physical-to-virtual transmission, modeling, and decision-making, to evaluate digital twin maturity levels. Overall, existing research primarily focuses on the theoretical framework for assessing the maturity of digital twins (DT). However, there is still no consensus on the precise definition of maturity stages. Additionally, compared to theoretical studies, empirical research based on case studies remains relatively underdeveloped, limiting a deeper understanding of DT application maturity.

In summary, although various frameworks and perspectives for evaluating digital twin maturity have been proposed in the construction field, significant challenges remain:

(1) **Lack of Standardization:** The construction phase of the industry lacks standardized definitions, application frameworks, and maturity models for digital twins [44].
(2) **Limitations of Traditional Evaluation Methods:** Traditional evaluation methods are predominantly qualitative, characterized by a high degree of subjectivity. Even quantitative approaches suffer from limited intuitiveness and inadequate capacity to handle uncertainty and complex decision-making. Furthermore, the absence of a scientifically sound and well-structured feedback mechanism hampers the formulation of objective and precise improvement recommendations.
(3) **Insufficient Data and Case Studies:** Limited data related to the construction phase and a lack of case studies impede practical research on digital twin maturity assessment.

**Fig 1:**
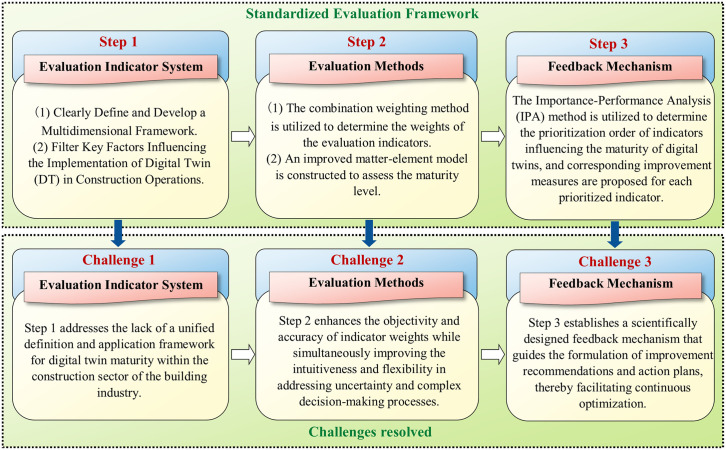
A standardized framework for assessing the maturity of digital twins during the construction phase.

To address these gaps, this study aims to systematically explore the evaluation of digital twin maturity during the construction phase, thereby promoting the application of digital twin technology in construction activities. For this purpose, we developed a standardized evaluation framework specifically designed for assessing digital twin maturity during the construction phase (Fig 1). Subsequently, this framework was applied to real construction projects, enriching the domain of practice-oriented case studies.

## 2. Research methods

The evaluation process for digital twin maturity encompasses three integral components: the establishment of a comprehensive evaluation indicator system, the determination of robust evaluation methodologies alongside clearly defined grade standards, and the development of systematic feedback mechanisms.

### 2.1 Evaluation indicator system

Building upon existing research definitions [42,43], this study posits that, in the construction phase, the maturity of digital twins refers to the capability level achieved through real-time dynamic mapping between virtual models and physical construction sites. This maturity is characterized by data-driven resource optimization, risk prediction, and collaborative management. The central objective is to transition from reactive responses to proactive interventions, ultimately establishing a closed-loop optimized intelligent construction system.

**Fig 2:**
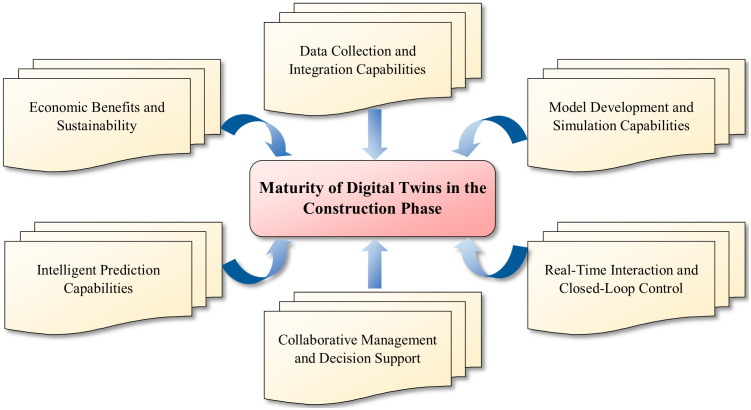
Key factors influencing the maturity of digital twins in the construction phase.

Additionally, considering existing interpretations of digital twin maturity within the construction industry [45], this study identifies several critical factors for evaluating the maturity of digital twins in the construction phase. These factors include data collection and integration capabilities, model development and simulation capabilities, real-time interaction and closed-loop control, collaborative management and decision support, intelligent prediction capabilities, as well as economic benefits and sustainability (Fig 2).

#### 2.1.1 Evaluation Indicators.

Given the unique characteristics of digital twin technology during the construction phase, this study builds upon the traditional three-dimensional digital twin model (physical, virtual, and connected) [45] and proposes five refined dimensions for maturity assessment: the acquisition layer, data layer, modeling layer, analysis layer, and application layer (refer to Table 1).

**Table 1:** Specific meanings of digital twin maturity assessment dimensions.

Maturity Dimensions	Index Number	Interpretation
Acquisition Layer	A	The acquisition layer captures the real-time state of construction sites. By deploying various sensors in buildings and environments, it collects physical environmental data, including geometry, materials, performance, and historical records. This layer comprises a perception module for data collection and a network module for transmitting data to virtual spaces.
Data Layer	B	The data layer serves as the foundational element for implementing interactive services within Digital Twin systems. During the process of data collection from construction sites, substantial amounts of multi-source heterogeneous data are generated. Consequently, stringent requirements are imposed on the quality of twin data, as well as on its transmission, storage methods, security, real-time processing capabilities, and visualization.
Modeling Layer	C	The modeling layer functions as the process by which a “digital mirror” accurately represents physical entities. By conducting geometric modeling within virtual space, it captures geometric information such as dimensions, size, shape, and spatial relationships of the physical environment. Subsequently, the collected physical attribute data is integrated with the three-dimensional model, establishing an interactive connection between the physical space and the virtual model.
Analysis Layer	D	The analysis layer facilitates the real-time collection and integration of vast amounts of multi-source heterogeneous raw data from both physical and virtual spaces through IoT sensing technologies. By employing intelligent algorithms, the data is subjected to mining and analytical processes to achieve classification and prediction outcomes. Ultimately, the powerful data-processing capabilities of the algorithm library in the virtual space are leveraged to support decision-making and optimization.
Application Layer	E	The application layer, as a service system founded on Digital Twin technology, integrates information from the construction phase through the establishment of a Digital Twin platform. By utilizing functional modules such as safety management and production management, it enables visualization of the construction process and facilitates resource sharing, ultimately achieving digital management and efficient control of construction activities.

#### 2.1.2 Evaluation sub-dimensions.

Using keywords such as “Digital Twin Technology,” “Digital Twin Maturity Assessment,” and “Digital Twin Construction,” a literature search was conducted in the Web of Science and Scopus databases. A total of 102 academic papers and 12 doctoral dissertations were selected for detailed review and synthesis. Additionally, using “Digital Twin” as the search term, 70 relevant items and documents were retrieved from the official website of the Ministry of Housing and Urban-Rural Development, with the search timeframe set to August 1, 2024. Following an analysis of these research outcomes and policy documents, 35 maturity evaluation indicators across five dimensions were identified. However, redundant information within some indicators posed potential challenges to the accuracy of evaluation results, necessitating further refinement. The Delphi method was employed to filter these indicators [46]. Specifically, 20 experts—including six academic scholars, seven construction management professionals, and seven technical specialists from Digital Twin platform providers—were invited to assess the rationality of the initially proposed 35 indicators on a scale from 1 to 10. These experts, confirmed prior to the survey to possess substantial knowledge of Digital Twin research, responded positively to the evaluation. The selection of indicators was based on the level of consensus among the experts, as measured by Kendall’s coefficient of concordance (W). Indicators were retained if the significance test of W yielded P < 0.05 [46]. Statistical analysis was conducted using IBM SPSS software. Indicators with p-values greater than or equal to 0.05 were excluded, resulting in a final set of 30 evaluation indicators. Details of the excluded indicators are presented in Table 2.

**Table 2:** Summary of excluded indicators.

Excluded Indicator	Maturity Dimensions	Expert-Rated Reason for Exclusion
Construction log text analysis capability (p = 0.15)	Analysis Layer	Redundant with other structured data analysis; limited applicability in real-world scenarios
Equipment QR code recognition rate (p = 0.24)	Acquisition Layer	High technological maturity and low variability; insufficient to reflect system capability
Aesthetic quality of data visualization (p = 0.38)	Application Layer	Highly subjective and difficult to quantify
Model rendering speed (p = 0.17)	Modeling Layer	Primarily influenced by hardware performance; not a core indicator of system maturity
Number of cloud platform integrations (p = 0.22)	Data Layer	Quantity does not directly reflect data quality or management capability

To mitigate redundancy among indicators, repeated or overlapping indicators were eliminated, and those with identical meanings were merged. The main steps for indicator screening were as follows:

(1) Regression Analysis: Each preliminary indicator was subjected to regression analysis, using other indicators as predictor variables and the given indicator as the response variable. The coefficient of determination (*R²*) for each regression model was calculated, with the entire process conducted in IBM SPSS software.
(2) Variance Inflation Factor (VIF) Calculation: The variance inflation factor (VIF) was computed using the formula [43]:

$$VIF=\text{1}/(\text{1}-x^{\text{2}})$$ (1)

Where *R* represents the correlation among all indicators within each driving factor. Typically, indicators are considered to exhibit multicollinearity if *VIF* > 10, whereas 0 ≤ *VIF* ≤ 10 indicates the absence of multicollinearity [47].

Highly correlated indicators were eliminated using the Variance Inflation Factor (VIF) method, as detailed in Table 3. Following this refinement, a total of 26 evaluation indicators were retained (see Table 4).

**Table 3:** Summary of highly correlated indicators removed based on VIF analysis.

Correlated Indicators	Removed Indicator	Maturity Dimensions	VIF	Justification for Exclusion
Video surveillance integration & sensor modality	Video surveillance integration	Acquisition Layer	27	Video surveillance is a subset of sensor modality and is therefore already represented
Data interface openness & data transmission	Data interface openness	Data Layer	19	Interface openness reflects the technical implementation of data transmission; high redundancy
Model lightweighting capability & model visualization capability	Model lightweighting capability	Modeling Layer	32	Lightweighting is a technical means of visualization and thus already encompassed
Automatic anomaly detection rate & analytical capability	Automatic anomaly detection rate	Analysis Layer	39	Anomaly detection is a specific application of analytical capability and is therefore subsumed

**Table 4:** Digital twin maturity evaluation indicators.

Maturity dimension	Index Number	Maturity indicators	References
Acquisition Layer A	A_1_	Sensing forms	[6], [48]
	A_2_	Sensing capability	[49], [50,51]
	A_3_	Network infrastructure	[52], [53]
Data Layer B	B_1_	Data transmission	[54], [55]
	B_2_	Data quality	[56], [31,57]
	B_3_	Data storage	[58]
	B_4_	Data timeliness	[59], [60]
	B_5_	Data security	[61]
	B_6_	Data visualization	[62], [63]
Modeling Layer C	C_1_	Model accuracy	[39]
	C_2_	Model regularization	[29]
	C_3_	Model synchronization capability	[56], [31]
	C_4_	Model visualization capability	[39]
	C_5_	Model information integration capability	[56], [39]
Analysis Layer D	D_1_	Analytical capability	[20]
	D_2_	Decision-making capability	[50], [64]
	D_3_	Predictive capability	[65], [66,67]
	D_4_	Optimization capability	[68], [69]
	D_5_	Autonomous learning capability	[70], [71]
Application Layer E	E_1_	Production management capability	[72], [73]
	E_2_	Equipment management capability	[74], [75]
	E_3_	Material management capability	[74], [75]
	E_4_	Quality management capability	[20]
	E_5_	Safety management capability	[20], [72,73]
	E_6_	personnel management capability	[75], [76]
	E_7_	Technical management capability	[21], [77]

### 2.2 Evaluation methods and levels

This study initially adopts a combined AHP-entropy weight method to assign weights to each dimension level. The AHP method is utilized to determine the subjective weights of dimensions and indicators, while the entropy weight method is applied to calculate objective weights. Finally, the comprehensive weights are computed through the product normalization method. Subsequently, the matter-element model is employed, integrating expert scoring results with comprehensive weight results to derive the comprehensive correlation degree of the evaluated objects, thereby determining the evaluation level.

#### 2.2.1 Determination of indicator weights.

(1) Subjective Weight Calculation—AHP

Step 1: Constructing the Judgment Matrix The judgment matrix reflects the relative importance of each element within the evaluation layer concerning a particular element in the higher layer. Its mathematical expression is as follows:


$$X=[\begin{array}{ccccc} X_{\text{1}\text{1}} & X_{\text{1}\text{2}} & X_{\text{1}\text{3}} & ... & X_{\text{1}n}\\ X_{\text{2}\text{1}} & X_{\text{2}\text{2}} & X_{\text{2}\text{3}} & ... & X_{\text{2}n}\\ X_{\text{3}\text{1}} & X_{\text{3}\text{2}} & X_{\text{3}\text{3}} & ... & X_{\text{3}n}\\ ... & ... & ... & ... & ...\\ X_{n\text{1}} & X_{n\text{2}} & X_{n\text{3}} & ... & X_{nn} \end{array}]$$ (2)

For any judgment matrix, the following conditions must be satisfied: *X*__*ij*__ = 1 and $X_{ij}=\frac{\text{1}}{X_{ji}}$. *X*__*ij*__ represents the judgment value of the relative importance of element *X*__*i*__ to *X*__*j*__. The graded standard values of *X*__*ij*__ are determined based on Saaty’s 1–9 scale method (see Table 5).

**Table 5:** Saaty’s 1–9 scale method.

Scale Value	Meaning
1	Equally important
3	Slightly more important
5	Clearly more important
7	Much more important
9	Absolutely more important
2, 4, 6, 8	Intermediate between adjacent levels

Step 2: Conducting Hierarchical Single Sorting

1) Calculate the product of the elements in each row of the judgment matrix, denoted as *M*_*i*_:

$$M_{i}=\prod {\mathrm{\backslash nolimits}}_{j=\text{1}}^{n}X_{ij}$$ (3)

2) Compute the Nth root of *M*, denoted as *W*__*i*__.
3) Normalize *W*__*i*__ to obtain the weight of the indicators, denoted as *Z*__*i*__, using the following formula:

$$Z_{i}=\frac{W_{i}}{\sum_{i=\text{1}}^{n}W_{i}}$$ (4)

Step 3: Conducting Consistency Checks on the Matrix

1) Calculate the maximum eigenvalue (𝜆*max*) of each judgment matrix.
2) Compute the consistency index (CI) using the formula:

$$CI=\frac{\lambda_{\text{max}}-n}{n-\text{1}}$$ (5)

The random consistency index (RI) is related to the order of the judgment matrix (see Table 6). Generally, as the order of the matrix increases, the likelihood of random deviations in consistency becomes higher.

**Table 6:** Standard values of the average Random Consistency Index (RI).

The order	1	2	3	4	5	6	7	8	9
RI	0	0	0.58	0.89	1.12	1.26	1.36	1.41	1.46

3) Calculate the consistency ratio (CR) using the formula:

$$CR=\frac{CI}{RI}$$ (6)

If *CR* < 0.10, the matrix is considered to meet the consistency requirement. If *CR* > 0.10, adjustments to the judgment matrix are required until the condition is satisfied.

(2) Objective Weight Calculation

Entropy Weight Method The entropy weight method determines weights based on the degree to which each evaluation indicator influences the overall system. The specific calculation steps are as follows:

Step 1: Forming the Original Data Sequence Matrix

For the given evaluation objects *M*__*i*__ (_*i*_ = 1, 2, 3, 4, …, *m*) and evaluation indicators *N*_*f*_ (*f* = 1, 2, 3, 4, …, *n*), the evaluation value of object *M*__*i*__ under indicator *N*_*f*_ is denoted as *R*__*i*_*f*_ (_*i*_ = 1, 2, 3, … ,*m*; *f* = 1, 2, 3, …, *n*). This forms the matrix *R*.


$$R={\left[\begin{array}{cccc} r_{11} & r_{12} & ... & r_{1n}\\ r_{21} & r_{22} & ... & r_{2n}\\ ... & ... & ... & ...\\ r_{m1} & r_{m1} & ... & r_{mn} \end{array}\right]}_{mn}$$ (7)

Step 2: Normalizing the Original Matrix

The original matrix is normalized to ensure that *V*__*i*_*f*_ falls within the range of 0–1. In this study, the threshold method (also known as the critical value method) is employed for processing indicators where “the larger, the better.”


$$V_{if}=\frac{r_{if}-\text{min}\left(r_{f}\right)}{\text{max}\left(r_{f}\right)-\text{min}\left(r_{f}\right)}$$ (8)

Step 3: Calculating the Proportion of Indicator Values for Object *i* under Indicator *f*, *P*__*i*_*f*_


$$P_{if}=\frac{V_{if}}{\sum_{i=\text{1}}^{m}V_{if}}$$ (9)

Step 4: Calculating the Entropy Value of Indicator *f*


$$e_{f}=\frac{-\text{1}}{\text{ln}(m)\sum_{i=\text{1}}^{m}P_{if}\times \text{ln}P_{if}}$$ (10)

Step 5: Calculating Entropy Weight *Z*_*f*_

The divergence coefficient *d*_*f*_ is introduced, defined as *d*_*f *_= 1 − *ef*. A larger *d*_*f*_ implies greater divergence for the corresponding indicator, indicating that it carries more information and should be assigned a higher weight. The formula for calculating entropy weight *Z*_*f*_ is as follows:


$$Z_{f}=\frac{d_{f}}{\sum_{f=\text{1}}^{n}d_{f}}$$ (11)

(3) Comprehensive Weight Calculation

In the process of determining weights by combining the Analytic Hierarchy Process (AHP) and the entropy weight method, this approach not only takes expert experience into account but also reduces the influence of subjective factors on the results. This allows for a more objective and reasonable determination of the weights for each evaluation indicator.

The formula for calculating the comprehensive weight *W*_*j*_ is as follows:


$$W_{j}=\frac{W_{AHP}W_{Entropy}}{\sum_{j=\text{1}}^{n}W_{\text{AHP}}W_{Entropy}}$$ (12)

#### 2.2.2 Construction of the optimized matter-element model.

The matter-element model, originally proposed by Chinese mathematician Wen Tsai in the 1980s, provides an effective method to resolve incompatibility issues in complex systems [78]. This model encompasses three fundamental steps: defining the classical and joint domains, establishing the correlation function, and calculating the comprehensive correlation degree [78]. As detailed in Section 2.2.1, combining the Analytic Hierarchy Process (AHP) with entropy weighting was utilized to derive indicator weights, thereby reducing subjectivity in scoring to some extent. However, traditional models still exhibit the following limitations:

(1) Classical domains often rely on fixed thresholds, potentially causing abrupt changes at correlation boundaries that lead to unreasonable evaluation grades (e.g., while scores of 0.79 and 0.81 are numerically close, their grade differences may be stark).
(2) Correlation functions are piecewise linear, which may introduce inaccuracies due to potential function distortions.

**Fig 3:**
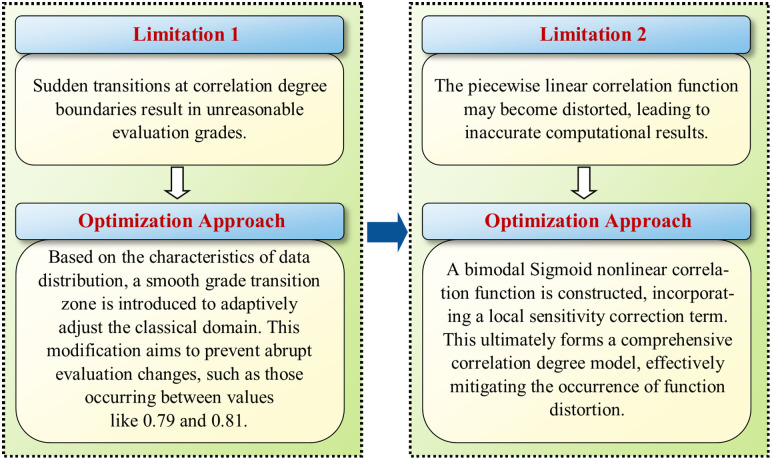
Optimization process of the traditional matter-element model.

To overcome the two aforementioned limitations, we have improved the traditional matter-element model, as detailed in Fig 3.

The optimized matter-element model involves the following steps:

Step 1: Define the Matter-Element Structure

The matter-element structure is defined as:


$$R=(N,C,V)=\left\{ \;\;\;\;\begin{array}{c} N:\text{The object being evaluated}\\ C=\left\{c_{\text{1}},c_{\text{2}},\ldots ,c_{m}\right\}:\text{The set of evaluation indicators}\\ V=\left\{v_{\text{1}},v_{\text{2}},\ldots ,v_{k}\right\}:\text{The set of grades} \end{array}\right.\;$$ (13)

Step 2: Define Classical and Joint Domains

(1) Classical Domain Matrix A:

$$A=(\begin{array}{ccc} \left[a_{\text{1}\text{1}}^{-},a_{\text{1}\text{1}}^{+}\right] & \cdots & \left[a_{\text{1}K}^{-},a_{\text{1}K}^{+}\right]\\ \vdots & \ddots & \vdots \\ \left[a_{m\text{1}}^{-},a_{m\text{1}}^{+}\right] & \cdots & a_{mK}^{-},a_{mK}^{+} \end{array})$$ (14)

Here, $a_{jk}^{-}$ and $a_{jk}^{+}$ indicate the lower and upper threshold values of indicator $c_{j}$ within grade $v_{k}$, respectively.

(2) Joint Domain $\;X_{j}$:

$$X_{j}=\left[\underset{k}{\text{min}}\;a_{jk}^{-},\underset{k}{\text{max}}\;a_{jk}^{+}\right],\;j=\text{1},\ldots ,m$$ (15)

Step 3: Dynamic Threshold Optimization

(1) The classical domain is adjusted adaptively using the following formula:

${\tilde{a}}_{jk}^{-}=a_{jk}^{-}-\delta_{j}$,${\tilde{a}}_{jk}^{+}={\tilde{a}}_{jk}^{+}+\delta_{j}$ (16)

In this context, $\delta_{j}$ denotes the expansion amplitude. Existing studies suggest that the formulation is considered most appropriate for boundary adjustment in maturity evaluation models [79] (See S1 Appendix in the Supporting Information for details).

Based on expert scoring data collected in this study, six candidate values were comparatively analyzed: 𝛿_*j*_ = 0.05∣*X*_*j*_∣, 𝛿_*j*_ = 0.06|*X*_*j*_|, 𝛿_*j*_ = 0.07|*X*_*j*_|, 𝛿_*j*_ = 0.08|*X*_*j*_|, 𝛿_*j*_ = 0.09|*X*_*j*_|, and 𝛿_*j*_ = 0.10|*X*|. The evaluation focused on misclassification rates of maturity levels, boundary sensitivity, and the maximum correlation degree (See S2 Appendix in the Supporting Information for details). The results indicate that when 𝛿_*j*_ = 0.07|*X*_*j*_|, the boundary misclassification rate is minimized (0%), and a favorable balance is achieved between sensitivity and stability, with a more pronounced maximum correlation degree. These findings support the adoption of 𝛿_*j*_ = 0.07|*X*_*j*_| as the optimal parameter for the evaluation model in this project.

(2) The standard distance is then computed using the formula:

$$d_{jk}=\frac{x_{j}-\mu_{jk}}{\sigma_{jk}}$$ (17)

Here: *x*__*j*__: Normalized indicator score, 𝜇__*j*_*k*_: Adjusted grade center, calculated as $\mu_{jk}=\frac{{\tilde{a}}_{jk}^{-}+{\tilde{a}}_{jk}^{+}}{\text{2}}$, 𝜎__*j*_*k*_: Adjusted grade bandwidth, calculated as $\sigma_{jk}=\frac{{\tilde{a}}_{jk}^{-}-{\tilde{a}}_{jk}^{+}}{\text{4}}$.

Step 4: Nonlinear Correlation Degree Calculation

(1) The nonlinear correlation degree *K*__*j*__(*v*_*k*_) is calculated using the bimodal Sigmoid correlation function as follows:

$$K_{j}\left(v_{k}\right)=\left\{ \begin{array}{c} \frac{\text{1}}{\text{1}+e^{-\beta_{\text{1}}(d_{jk}+\alpha )}}\;d_{jk}\leq \text{0}\;\\ \frac{\text{1}}{\text{1}+e^{-\beta_{\text{2}}(d_{jk}+\alpha )}}\;d_{jk}>\text{0} \end{array}\right.\;$$ (18)

Where: 𝛽_1_ = 8 and 𝛽_2_ = 5: Represent the decay rate parameters for the left and right sides, respectively [80]. 𝛼 = 0.3: Denotes the center offset [81].

(2) The local sensitivity correction term Δ*K*_*j*_(*v*_*k*_) is formulated as:

$$\Delta K_{j}\left(v_{k}\right)=\eta \left[\text{tan}h\left(\frac{x_{j}-{\tilde{a}}_{jk}^{-}}{\tau_{j}}\right)-\text{tan}h\left(\frac{x_{j}-{\tilde{a}}_{jk}^{+}}{\tau_{j}}\right)\right]$$ (19)

Where:$\;\tau_{j}=\frac{\sigma_{j}}{\text{3}}$: Represents the local bandwidth coefficient [82]. 𝜂 = 0.2: Denotes the correction intensity coefficient [83].

(3) The comprehensive correlation degree $\tilde{K_{j}\left(v_{k}\right)}$ is computed using the following formula:

$$\tilde{K_{j}\left(v_{k}\right)}=K_{j}\left(v_{k}\right)+\Delta K_{j}\left(v_{k}\right)$$ (20)

Step 5: Determination of Comprehensive Evaluation Grade

This study categorizes the application scenarios and functionalities of Digital Twin technology in the construction field into a hierarchical maturity framework consisting of five levels: (I) Basic, (II) Connected, (III) Integrated, (IV) Interactive, and (V) Autonomous. The proposed hierarchical structure represents a progressive classification based on the technological advancement and practical application of Digital Twin systems. Its objective is to assess the improvements in efficiency and advancements in technology. The corresponding score requirements and specific definitions for each level are provided in Table 7.

**Table 7:** Digital twin maturity evaluation levels.

Maturity levels	Name	Score range	Definition
Ⅰ	Basic level	[0, 60)	At this level, the Digital Twin is static, offering only limited and fundamental functionalities and services. A collaborative network has not been established, resulting in low efficiency in information communication and system management.
Ⅱ	Connected level	[60, 70)	At this level, the Digital Twin operates in a real-time and dynamic manner, capturing temporal characteristics. A one-way and persistent data connection from physical assets to the Digital Twin has been established. Additionally, a collaborative network and an efficient information communication system have been developed.
Ⅲ	Integrated level	[70, 80)	The Digital Twin evolves from isolated data collection and communication to integrated implementation, establishing bidirectional connectivity. Physical assets gain the capability to initiate feedback mechanisms, including remote control, real-time monitoring, and other maintenance functions.
Ⅳ	Interactive level	[80, 90)	At this stage, the Digital Twin demonstrates exceptional information richness, model accuracy, and predictive capabilities through interaction and feedback with physical entities.
Ⅴ	Autonomous level	[90, 100)	Advanced Digital Twins leverage methods such as machine learning and deep learning to interact and provide feedback with physical entities. They possess the capability for automated prediction and operation, as well as autonomous decision-making and optimization.

The correlation degree in the matter-element model extends the logical values from the [0,1] closed interval of fuzzy mathematics to the real number axis (−∞, +∞), which reveals more differentiation information:

(1) When 0≤ $\tilde{K_{j}\left(v_{k}\right)}$≤1, it indicates that the digital twin maturity level in this region meets the requirements of the standard level.
(2) When −1< $\tilde{K_{j}\left(v_{k}\right)}$ <0, it suggests that the level does not meet the standard requirements but still has the potential to belong to this interval. The closer its value is to zero, the more likely it is to be converted.
(3) When $\tilde{K_{j}\left(v_{k}\right)}$ ≤−1, it signifies that it is neither within this grade range nor has the conditions for conversion into this grade.
(4) When $\tilde{K_{j}\left(v_{k}\right)}$ >1, it indicates that the level exceeds the upper limit of the standard grade.

By integrating the previously determined indicator weights, the grade membership degree *D*_*k*_ can be calculated as follows:

The grade membership degree *D*_*k*_ is synthesized using the formula:


$$D_{k}=\sum_{j=\text{1}}^{m}w_{j}\tilde{K_{j}\left(v_{k}\right)}$$ (21)

Where: *w*__*j*__: Represents the weight of indicator _*j*_. $\tilde{K_{j}\left(v_{k}\right)}$: Denotes the comprehensive correlation degree for grade *v*_*k*_.

The maturity grade *L* and scoreis determined as:


$$L=arg\;\underset{k}{\text{max}}D_{k}$$ (22)

$$Score=\sum {\mathrm{\backslash nolimits}}_{k=\text{1}}^{K}D_{k}\times e^{\text{0}.\text{2}k}$$ (23)

### 2.3 Feedback mechanism

**Fig 4:**
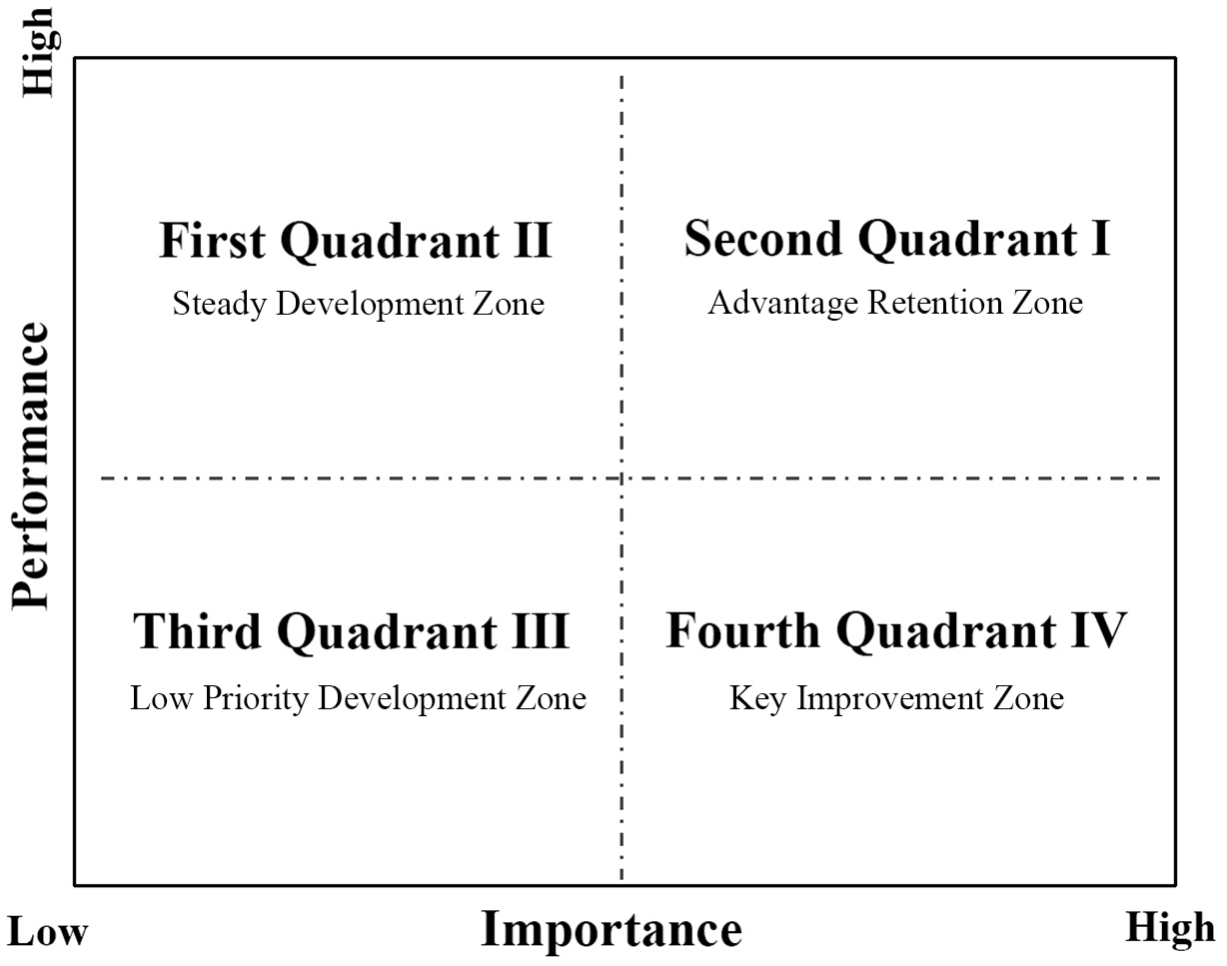
Schematic diagram of IPA quadrant partitioning.

The Importance-Performance Analysis (IPA), introduced by Martilla and James in 1977 [84], is a straightforward, intuitive, and visual evaluation method that has been extensively applied in the assessment of service quality across various domains [85]. This approach evaluates key elements from the dual perspectives of importance and performance, mapping the evaluation indicators across four quadrants based on their importance and performance levels, as demonstrated in Fig 4. In this study, the digital twin maturity evaluation employs weight values of indicators as the horizontal axis (importance) and maturity scores as the vertical axis (maturity). Using the mean values of these two dimensions as thresholds, the matrix is divided into four quadrants: Quadrant I (high importance, high maturity) represents the “Advantage Retention Zone,” Quadrant II (low importance, high maturity) constitutes the “Steady Development Zone,” Quadrant III (low importance, low maturity) corresponds to the “Low Priority Development Zone,” and Quadrant IV (high importance, low maturity) signifies the “Key Improvement Zone.” This methodology facilitates cross-dimensional analysis of indicators, offering improvement recommendations tied to maturity results and providing scientific guidance for evaluation processes.

### 2.4 Alignment and advantages over existing standards

By systematically reviewing the methodological foundations and applicable scopes of existing standards and models, this study clarifies the distinctive features and innovations of the proposed evaluation framework in terms of goal orientation, structural granularity, assessment methodology, construction-phase alignment, and complementary potential. The framework’s relationship with established principles and standards is characterized by translation, integration, and extension. A comparative analysis is presented across four dimensions: the Gemini Principles, ISO 19650, the CMMI framework, and representative academic literature.

The Gemini Principles emphasize governance values such as purpose and trust, including data quality, security, visualization, and access control [20,86]. This study translates these abstract principles into measurable indicators within the data and application layers. Through dynamic thresholding and weight adjustment, the framework balances privacy, security, and openness across varying contexts. While the Gemini Principles are non-quantitative and architecture-oriented, the proposed framework operationalizes them into an evaluable governance–technology coupling mechanism, facilitating project-level implementation.

ISO 19650 focuses on lifecycle information management, including data organization, exchange, versioning, and Common Data Environment (CDE) collaboration [87–89]. The indicators defined in this study—such as B1 to B6 (transmission, quality, storage, timeliness, security, visualization) and C2/C3 (standardization/synchronization)—serve as quantitative externalizations of the information management process. These indicators enable monitoring of CDE performance, fidelity in cross-organizational exchange, and timeliness. Unlike ISO 19650, which centers on process roles and compliance, the proposed framework bridges procedural conformity with performance quantification.

The CMMI framework provides process domains and maturity levels for aspects such as data quality, risk, measurement, and governance. However, it does not address the closed-loop applications specific to construction-oriented digital twins, such as equipment, materials, quality, safety, personnel, and technology [90–93]. This study maps these operational capabilities to performance-linked indicators (E1–E7) and employs Importance–Performance Analysis (IPA) to identify high-importance but low-performance gaps. In doing so, it advances from assessing process existence to evaluating process effectiveness, offering a scenario-specific quantitative extension of CMMI for construction digital twin applications.

Existing academic literature primarily emphasizes layered digital twin capabilities, core technologies (data–model–analysis–feedback), and semantic interoperability requirements [86,94]. The five-layer structure (A–E) adopted in this study aligns closely with these technical logics. However, the framework introduces quantifiable management-oriented indicators tailored to the construction phase, along with a novel evaluation methodology that integrates matter-element modeling, dynamic thresholds, and nonlinear correlation functions. These enhancements enrich the methodological toolkit for maturity assessment.

Table 8 presents a comparative summary of the proposed framework against the Gemini Principles, ISO 19650, CMMI, and academic models across five dimensions: goal orientation, structural granularity, assessment methodology, construction-phase alignment, and complementary potential.

**Table 8:** Framework alignment with standards and literature.

Dimension	Proposed Evaluation Framework	Gemini Principles	ISO 19650	CMMI Framework	Academic Literature
Goal Orientation	Quantifiable maturity assessment and optimization loop for construction phase	Governance and ethical guidance on purpose, trust, and function [86]	Lifecycle information management and CDE coordination [87,89]	Organizational process capability and continuous improvement [90 − 92]	Capability profiling and key technology stack [20,86,94]
Structural Granularity	Five-layer decomposition (Acquisition/Data/Modeling/Analysis/Application) with refined indicators [20,94]	Principle-level, non-quantitative [86]	Concept–process–role–exchange; non-scoring [87,89]	Process domains and levels; not scenario-specific [90,91]	Layered capabilities/types; limited granularity for construction [20,94]
Assessment Methodology	Enhanced matter-element model with dynamic thresholds and nonlinear correlations; IPA feedback [93]	Principle compliance [86]	Process and role compliance [87,89]	Maturity level identification (ML1–ML5) [90–92]	Primarily qualitative; partial capability mapping [20,86,94]
Construction Alignment	High (covers equipment, materials, quality, safety, personnel, and technology management) [92])	Medium (requires indicator-level translation) [86]	Medium (requires mapping to construction KPIs) [95,96]	Medium (requires translation to construction performance) [90,91]	Medium (requires mapping to construction processes and KPIs) [20,94]
Complementary Potential	Translates principles/standards/process maturity into quantifiable indicators and weights [20,93]	Provides governance values and principles of openness/security [86]	Offers CDE/information exchange and role coordination [87,89]	Provides organizational improvement paths and management practices [90 − 92]	Offers capability layers and terminology consensus [20,86,94]

## 3. Case study

### 3.1 Project overview

#### 3.1.1 Project introduction.

**Fig 5:**
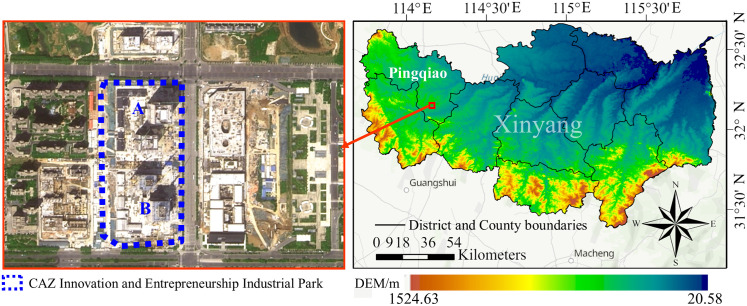
Location Map of Xinyang CAZ Innovation and Entrepreneurship Industrial Park. Note: The satellite basemap on the left is sourced from the official Tianditu website of China (https://www.tianditu.gov.cn/), while the basemap on the right is derived from USGS (viewer.nationalmap.gov/viewer).

The A and B buildings of the CAZ Innovation and Entrepreneurship Industrial Park in Xinyang, Henan Province, are currently the tallest landmark structures in the city. They are located in Pingqiao District, an area characterized by relatively flat terrain (as depicted in Fig 5). The project covers a land area of 39,685 square meters, with a total construction area of approximately 215,100 square meters. It comprises two tower buildings (Buildings A and B) and an associated commercial podium, with a standard floor height of 4.3 meters.

Despite facing constraints such as a tight construction schedule and significant technical challenges, the project team effectively employed advanced technologies, including Building Information Modeling (BIM) three-dimensional modeling, intelligent network platforms, and Eagle Eye panoramic monitoring systems, throughout the construction process. Moreover, the adoption of digital twin technology enabled the comprehensive digitization of physical structures, production elements, and management processes.

This project has been recognized as a “Digital Twin Intelligent Construction Site Demonstration Project” by Henan Province and serves as the first high-rise building in Xinyang to utilize digital twin technology for construction monitoring and management. Additionally, it represents a seminal example of digital twin applications in the construction sector across China.

#### 3.1.2 Overview of digital twin application in the project.

**Fig 6:**
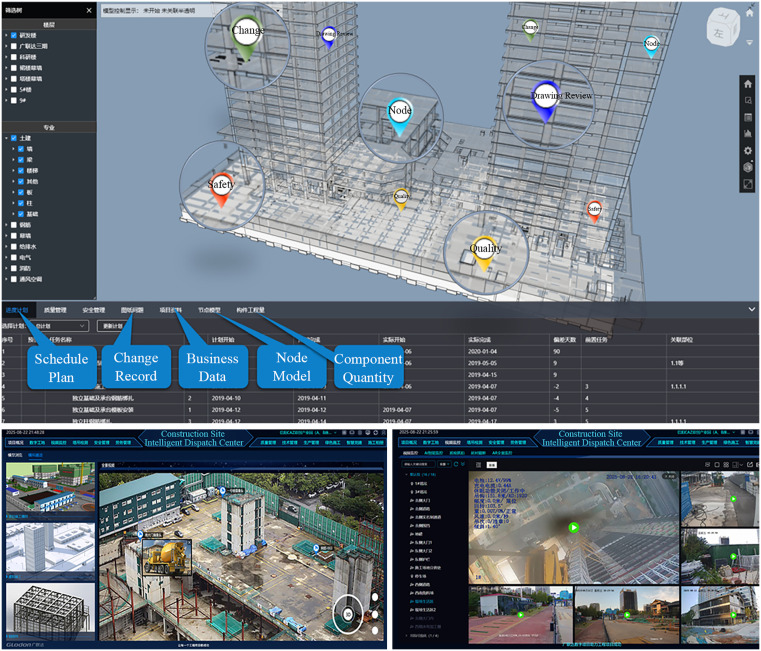
Illustration of the digital twin platform of the enterprise.

The project introduced an intelligent digital twin scheduling platform—referred to as the “Project Brain” (see Fig 6)—which enables real-time data aggregation, production tracking, and proactive risk mitigation. The system has been applied across multiple domains, including production management, equipment management, logistics, quality control, safety supervision, personnel coordination, and technical oversight. It has established a foundational level of usability in multi-source data acquisition, low-latency transmission, automated model interaction, platform governance, and scalability, thereby supporting continuous visualization, traceability, and predictive capabilities on-site. Key technical parameters are summarized in Table 9.

**Table 9:** Key technical parameters of the digital twin system.

Category	Key Parameter	Measured Value
Data Timeliness & Synchronization	End-to-end latency (mean/ 95th percentile)	150 s/ 240 s
	Visualization refresh cycle	0–60 s
	Time sync method/ accuracy	NTP (10–50 ms)
Model Accuracy & 4D Verification	Point cloud mean deviation/ 95th percentile	32 mm/ ~ 45–50 mm
	Component recognition F1 (independent test)	0.75
	4D auto-verification coverage (by quantity)	70%
	Point cloud/image acquisition cycle (key zones)	Every 2 weeks
Sensing & Deployment	UWB anchor density/ update rate	4 anchors per 600–800 m²; 5 Hz
	UWB horizontal error (mean/ 95th percentile)	0.4 m/ 0.8 m
	Video resolution/ frame rate/ edge inference delay	1080p @ 15 fps/ 800–1200 ms
	RFID channels/ read rate	2 channels/ 400 tags/s
Network & Edge Computing	5G/private network/ local Wi-Fi latency	10–30 ms/ 20–50 ms
	Edge coverage (for safety/location inference)	30% of cameras
Platform Performance	Real-time throughput (messages/sec, project level)	~800–1200
	Data loss rate	5%
Material Management KPIs	OTIF (On-Time In-Full)	88–90%
	Inventory accuracy (book-to-actual match)	92–94%
	Downtime due to material issues	5–6%
	Location/traceability loss rate	10–12%
Forecasting & Calibration	Short-term schedule forecast/ rolling calibration	3 days/ every 2 weeks
Safety Alerts	Violation/boundary alert latency (95th percentile)	20–30 s
Quality/Process Alerts	End-to-end latency (95th percentile)	10–20 min
Cost Targets	Construction CAPEX (contract ratio)	0.85%
	Annual OPEX (contract ratio)	0.15%

The data acquisition framework integrates Ultra-Wideband (UWB) anchors, video feeds, RFID tags, point cloud and image data, and Network Time Protocol (NTP) synchronization. This multi-source configuration supports localization, monitoring, and temporal alignment, enabling stable tracking of personnel and materials in medium-scale construction environments. It facilitates parallel acquisition of structured and unstructured data, laying a solid foundation for multi-model fusion. However, improvements in positioning accuracy and update frequency remain necessary.

In terms of data processing, the system leverages 5G/private networks and local Wi-Fi to achieve low-latency transmission and a complete end-to-end processing pipeline, encompassing acquisition, transmission, computation, and visualization. Fault tolerance and redundancy mechanisms are in place to ensure continuous operation, though further optimization is needed under high-concurrency conditions, particularly in cross-source temporal alignment.

Model interaction capabilities are reflected in geometric accuracy (mean deviation of 32 mm), component recognition performance (F1 score of 0.75), and 4D verification coverage (70%). The system supports stable visualization and alerting for situational awareness and post-event review, with strong human–machine collaboration features that allow rapid manual correction in complex scenarios. Nonetheless, reducing alert response time could enhance real-time responsiveness.

Platform governance and analytical capabilities are well-developed, with clear budget allocation (CAPEX 0.85%, OPEX 0.15%) and a KPI system that supports scalability to larger or multi-regional projects. Enhancing material traceability and improving On-Time In-Full (OTIF) performance would further strengthen operational efficiency.

### 3.2 Evaluation of digital twin maturity

#### 3.2.1 Determination of weights.

A panel of 20 experts was invited to score each indicator based on the actual conditions of the project, with questionnaire scores ranging from 0 to 100. The panel included six academic scholars specializing in digital twin applications in architecture, seven management personnel from construction enterprises, and seven technical staff members from digital twin platform providers. All experts responded positively, resulting in a 100% valid questionnaire recovery rate.

The combined weight of each indicator was calculated using the Analytic Hierarchy Process (AHP)-entropy weight method, as shown in Table 10. It was observed that among the primary indicators, the “Analysis Layer” and the “Application Layer” ranked the highest in weight, highlighting their significant impact on the digital twin maturity during the construction process. Among the secondary indicators, “Self-Learning Capability,” “Predictive Capability,” “Production Management Capability,” “Quality Management Capability,” and “Model Visualization Capability” emerged as the top five, emphasizing their crucial contributions to digital twin maturity at this stage.

**Table 10:** Combined weight results of evaluation indicators.

First-level Indicators	Weight	Second-level Indicators	Subjective Weight	Objective Weight	Combined Weight	Overall Weight
Collection Layer (A)	0.0523	Sensor Type (A_1_)	0.5396	0.34	0.5396	0.0282
		Sensor Capability (A_2_)	0.297	0.51	0.297	0.0155
		Network Infrastructure (A_3_)	0.1634	0.15	0.1634	0.0085
Data Layer (B)	0.1143	Data Transmission (B_1_)	0.071	0.1	0.0711	0.0081
		Data Quality (B_2_)	0.443	0.22	0.4434	0.0507
		Data Storage (B_3_)	0.229	0.07	0.2292	0.0262
		Data Real-time Capability (B_4_)	0.144	0.21	0.1444	0.0165
		Data Security (B_5_)	0.044	0.21	0.0437	0.005
		Data Visualization (B_6_)	0.068	0.19	0.0682	0.0078
Modeling Layer (C)	0.1878	Model Accuracy (C_1_)	0.05	0.13	0.0504	0.0095
		Model Standardization (C_2_)	0.076	0.14	0.0763	0.0143
		Model Synchronization Capability (C_3_)	0.173	0.23	0.1731	0.0325
		Model Visualization Capability (C_4_)	0.426	0.34	0.4258	0.08
		Model Integration Capability (C_5_)	0.274	0.15	0.2744	0.0515
Analysis Layer (D)	0.3246	Analytical Capability (D_1_)	0.04	0.07	0.0404	0.0131
		Decision-making Capability (D_2_)	0.176	0.05	0.1759	0.0571
		Predictive Capability (D_3_)	0.277	0.09	0.2765	0.0898
		Optimization Capability (D_4_)	0.08	0.05	0.0804	0.0261
		Self-learning Capability (D_5_)	0.427	0.73	0.4267	0.1385
Application Layer (E)	0.321	Production Management Capability (E_1_)	0.268	0.23	0.2685	0.0862
		Equipment Management Capability (E_2_)	0.099	0.17	0.099	0.0318
		Material Management Capability (E_3_)	0.04	0.29	0.0397	0.0127
		Quality Management Capability (E_4_)	0.268	0.11	0.2685	0.0862
		Safety Management Capability (E_5_)	0.165	0.12	0.1651	0.053
		Personnel Management Capability (E_6_)	0.099	0.04	0.099	0.0318
		Technical Management Capability (E_7_)	0.06	0.04	0.0603	0.0194

In contrast, the Collection Layer received the lowest weight among the primary indicators, suggesting that its influence on digital twin maturity during the construction phase is relatively limited. Similarly, Data Security ranked lowest among the secondary indicators, indicating that its contribution to maturity in this context is also minimal. In the specific case examined, experts justified the reduced weighting of data security by noting that many digital twin systems deployed on Chinese construction sites operate within local area networks, enterprise intranets, or private 5G environments. These configurations significantly limit exposure to external internet connections, thereby reducing the risk of external cyberattacks [97].

#### 3.2.2 Maturity evaluation results.

Based on the scoring of the digital twin maturity evaluation indicators for the Xinyang CAZ Innovation and Entrepreneurship Industrial Park by 20 experts, the average score across all participants was calculated and adopted as the final evaluation score for each indicator (refer to Table 11).

**Table 11:** Initial data of the matter-element model.

First-level Indicator	Code	Second-level Indicator	Score
Collection Layer (A)	A_1_	Sensor Type	76.5
	A_2_	Sensor Capability	68.7
	A_3_	Network Infrastructure	80.1
Data Layer (B)	B_1_	Data Transmission	75.8
	B_2_	Data Quality	85.1
	B_3_	Data Storage	84.4
	B_4_	Data Real-time Capability	64.4
	B_5_	Data Security	73.7
	B_6_	Data Visualization	63
Modeling Layer (C)	C_1_	Model Accuracy	46.5
	C_2_	Model Standardization	78.7
	C_3_	Model Synchronization Capability	61.5
	C_4_	Model Visualization Capability	73
	C_5_	Model Integration Capability	71.5
Analysis Layer (D)	D_1_	Analytical Capability	70.1
	D_2_	Decision-making Capability	71.5
	D_3_	Predictive Capability	67.2
	D_4_	Optimization Capability	73
	D_5_	Self-learning Capability	65.8
Application Layer (E)	E_1_	Production Management Capability	78
	E_2_	Equipment Management Capability	73
	E_3_	Material Management Capability	50.1
	E_4_	Quality Management Capability	77.2
	E_5_	Safety Management Capability	80.8
	E_6_	Personnel Management Capability	90.1
	E_7_	Technical Management Capability	86.5

Based on the classification in Table 7 and the integration of the dynamic threshold optimization algorithm, the initial classical domain and adjusted classical domain for evaluating the maturity indicators of digital twins were identified (refer to Table 12). Subsequently, the linear and nonlinear correlations of the primary and secondary indicators were calculated using both traditional and optimized models, with the results detailed in Table 13. A comparison of the correlation degrees calculated by the two methods revealed that their levels were nearly identical. However, the correlation values derived from the optimized model were higher, providing a more intuitive representation of the correlation levels.

**Table 12:** Classical domain and node domain of digital twin maturity evaluation.

Level	Original Classical Domain	Adjusted Classical Domain	Semantic description	Adjusted grade center${/\mu }_{k}$	Adjusted grade bandwidth/$\sigma_{k}$	$\delta_{j}$
V	[90,100]	[1,83 00]	Autonomous Level	92.5	4.25	7
IV	[80, 90)	[73, 97)	Interactive Level	85	6	
III	[70, 80)	[63, 87)	Integrated Level	75	6	
II	[60, 70)	[53, 77)	Connected Level	65	6	
I	[0,60)	[0, 67)	Basic Level	33.5	16.75	

Note: Given that *δ*_*j*_ = 7, the adjusted classical domains for levels V and I should have been [83,107] and [−7, 67), respectively. However, to conform to the threshold range of [0, 100], the classical domains of levels V and I were clipped to [83,100] and [0, 67), respectively.

**Table 13:** Results of nonlinear correlations for primary and secondary evaluation indicators across different levels.

Type	Code	Traditional Model Levels	Optimization Model Levels	Traditional Association Degree (Corresponding Levels)	Optimized Association Degree (Corresponding Levels)	Correlation Improvement Magnitude
Primary Indicators	A	III	III	0.16 (III)	0.91(III)	+469%
	B	IV	IV	0.22 (IV)	0.88 (IV)	+300%
	C	III	III	0.17 (III)	0.86 (III)	+406%
	D	II	II	0.39 (II)	0.92 (II)	+136%
	E	III	III	0.13 (III)	0.90 (III)	+592%
Secondary Indicators	A_1_	III	III	0.35 (III)	0.91 (III)	+160%
	A_2_	II	II	0.13 (II)	0.89 (II)	+585%
	A_3_	IV	IV	0.01 (IV)	0.90 (IV)	+8900%
	B_1_	III	III	0.42 (III)	0.91(III)	+117%
	B_2_	IV	IV	0.49 (IV)	0.94 (IV)	+92%
	B_3_	IV	IV	0.44 (IV)	0.93 (IV)	+111%
	B_4_	II	II	0.44 (II)	0.92 (II)	+109%
	B_5_	III	III	0.16 (III)	0.88 (III)	+450%
	B_6_	II	II	0.30 (II)	0.93 (II)	+210%
	C_1_	I	I	−0.15 (I)	0.85 (I)	+667%
	C_2_	III	III	0.13 (III)	0.91 (III)	+600%
	C_3_	II	II	0.15 (II)	0.94 (II)	+527%
	C_4_	III	III	0.30 (III)	0.88 (III)	+193%
	C_5_	III	III	0.15 (III)	0.89 (III)	+493%
	D_1_	III	III	0.01 (III)	0.90 (III)	+8900%
	D_2_	III	III	0.15 (III)	0.89 (III)	+493%
	D_3_	II	II	0.28 (II)	0.93 (II)	+232%
	D_4_	III	II	0.30 (III)	0.88 (III)	+193%
	D_5_	II	II	0.42 (II)	0.91 (II)	+117%
	E_1_	III	III	0.20 (III)	0.90 (III)	+350%
	E_2_	III	II	0.30 (III)	0.88 (III)	+193%
	E_3_	I	I	−0.09 (I)	0.84 (I)	+1033%
	E_4_	III	III	0.28 (III)	0.90 (III)	+221%
	E_5_	IV	IV	0.08 (IV)	0.93 (IV)	+1063%
	E_6_	V	V	0.01 (V)	0.96 (V)	+9500%
	E_7_	IV	IV	0.35 (IV)	0.94 (IV)	+169%

Based on the calculated comprehensive correlation degrees of all evaluation indicators for the Xinyang CAZ Innovation and Entrepreneurship Industrial Park (see Table 14), the results indicate that the traditional matter-element model yields a maturity rating of Level III (Integrated), whereas the optimized model produces a lower rating of Level II (Connected). This discrepancy suggests that the digital twin technology applied during the construction phase of the project exhibits moderate maturity. Notably, the analytical layer demonstrates the lowest level of maturity, highlighting a critical area for improvement in data integration and model responsiveness.

**Table 14:** Digital twin maturity evaluation results.

Evaluation Object	Type	Indicator Relational Degree					Determined Level
		I (Basic)	II (Connected)	III (Integrated)	IV (Interactive)	V (Autonomous)	
Xinyang CAZ Innovation and Entrepreneurship Industrial Park	Traditional Model	0.01	0.27	0.45	0.27	0.01	III
	Optimization Model	0.32	0.89	0.63	0.48	0.22	II

#### 3.2.3 Analysis of evaluation results.

(1) Descriptive statistics of indicator maturity scores

**Fig 7:**
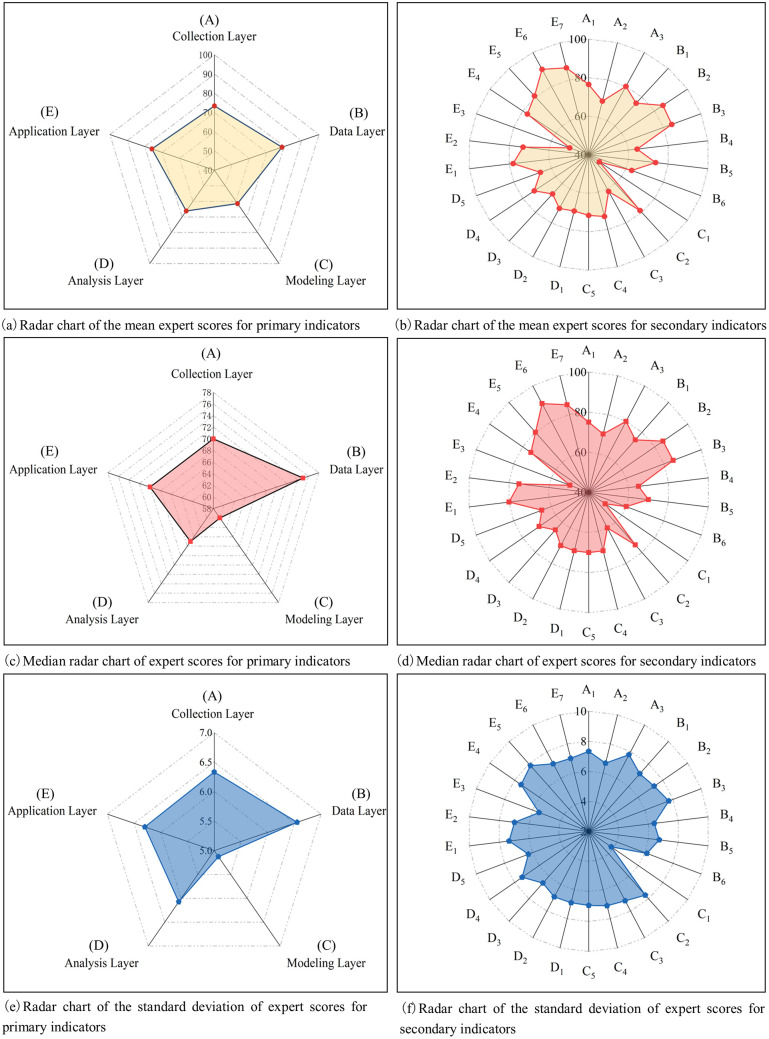
Descriptive statistical results of expert scores for evaluation indicators.

Based on the mean and median scores of the primary indicators evaluated by 20 experts (as shown in Fig 7(a) and 7(c)), the Modeling Layer (C) and Analysis Layer (D) had relatively lower mean scores and medians, ranking among the bottom two. This suggests that the interaction between the physical space and virtual models in this project is limited, and that the spatial algorithm library exhibits relatively weak capabilities in data processing, decision-making, and optimization. In contrast, the Data Layer (B) and Application Layer (E) achieved higher mean scores and medians, ranking among the top two, highlighting the superior quality, transmission efficiency, storage capability, and management effectiveness of the digital twin data during the construction process.

Furthermore, most secondary indicators showed mean and median scores ranging between 60 and 80 (as illustrated in Fig 7(b) and 7(d)). Among these, E_6_ (personnel management capability) achieved the highest mean and median scores, reflecting that the project’s high management standards are primarily attributable to effective personnel management. In contrast, the indicators ranked among the bottom two in terms of both mean and median scores were C_1_ (model accuracy) and E_3_ (material management capability), which fell below the passing threshold of 60. These findings highlight that low model accuracy and insufficient material management represent significant barriers to advancing the project’s digital twin maturity. In the specific case examined, two diagnostic findings support these conclusions. First, the low model accuracy is evidenced by an average deviation of 32 mm in point cloud alignment and component recognition. Prior research indicates that when this deviation consistently exceeds 30 mm, automated progress verification and quality deviation alerts—core functions of a self-aware and self-diagnostic digital twin—tend to regress into manual inspection and sampling. This transition effectively downgrades the system from a “digital twin” (characterized by high-fidelity bidirectional synchronization) to a “digital shadow” (unidirectional and low-trust) [98]. Second, construction records show that delays due to material mismatches, shortages, or omissions occurred four times during the project. Existing studies have demonstrated that material shortages and fluctuations in lead times can amplify schedule deviations and increase the risk of acceleration and rework, thereby distorting performance and inflating costs [95]. These factors explain the experts’ relatively low scores for model accuracy and material management capability.

As depicted in Fig 7(e) and 7(f), the standard deviations of the scores assigned by 20 experts to each indicator were consistently less than 10% of their respective mean values. This high level of consistency among expert evaluations further supports the reliability of the scoring data and bolsters the validity of the study’s conclusions.

(2) Analysis of Evaluation Levels

Based on the results in Table 13, it was observed that, for both primary and secondary indicators, the proportion of indicators with a correlation level of III was the highest (accounting for 60.00% and 46.15%, respectively). Furthermore, the overall maturity of the digital twin evaluation for this project was determined to be at Level II (Table 14). These findings suggest that the overall maturity level of the project’s digital twin technology is moderate, with considerable potential for enhancement in certain areas. Specifically:

1) Among the primary indicators, the relevance level of the data layer (B) ranks highest (Grade IV), while that of the analysis layer (D) is the lowest (Grade II). This suggests that the quality, transmission, and storage capabilities of the digital twin data are relatively high, whereas the data processing capacity of the spatial algorithm library and the overall decision-making and optimization capabilities are considerably weaker.
2) In the secondary indicators, personnel management capability (E6) achieved the highest correlation level (Level V), while eight indicators, including model accuracy (C1) and material management capability (E3), were evaluated at lower levels (Level II). This indicates that the personnel management level of digital twin technology in the project is relatively high. However, deficiencies in model accuracy and material management hinder the improvement of the project’s digital twin maturity.
3) Based on the analysis of primary and secondary indicators, the correlation levels of predictive capability (D3) and autonomous learning capability (D5) in the Analysis Layer (D) were relatively low, both rated at Level II. This indicates that deficiencies in intelligent event prediction and autonomous decision-making are key factors contributing to the weaker analytical capabilities of the digital twin technology in this project. Meanwhile, in the Data Layer (B), which has the highest maturity level, the secondary indicator of data timeliness (B4) was only rated at Level II. This reveals that, despite the overall high maturity of the Data Layer, there are still shortcomings in real-time data synchronization capabilities.

(3) Optimization Strategies for Feedback Evaluation

**Fig 8:**
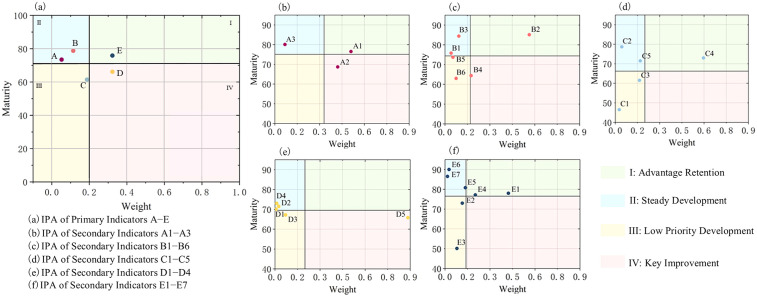
IPA chart of digital twin indicators (maturity−weight).

Through Importance-Performance Analysis (IPA) of primary and secondary indicators in the digital twin maturity evaluation, optimization strategies for feedback on project indicators have been determined, as illustrated in Fig 8(a)–8(f). The specific analysis is as follows:

1) Quadrant I (Advantage Retention Zone): This quadrant includes one primary indicator, the application layer (E), and five secondary indicators: sensing modalities (A_1_), data quality (B_2_), model visualization capability (C_4_), production management capability (E_1_), and quality management capability (E_4_). These indicators have generally met the expected standards of digital twin technology in the construction sector. They should remain well-maintained and continuously refined to sustain their performance.
2) Quadrant II (Steady Development Zone): This quadrant contains two primary indicators, the acquisition layer (A) and the data layer (B), along with eleven secondary indicators: network infrastructure (A_3_), data storage (B_3_), data transmission (B_1_), model standardization (C_2_), model-integrated information capability (C_5_), analytical capability (D_1_), decision-making capability (D_2_), optimization capability (D_4_), safety management capability (E_5_), personnel management capability (E_6_), and technical management capability (E_7_). These indicators have relatively lower impact on the construction sector’s digital twin technology but demonstrate high levels of development. As such, they require moderate attention without excessive resource allocation.
3) Quadrant III (Low Priority Development Zone): This quadrant includes one primary indicator, the modeling layer (C), and seven secondary indicators: data security (B_5_), data visualization (B_6_), model accuracy (C_1_), model synchronization capability (C_3_), prediction capability (D_3_), equipment management capability (E_2_), and material management capability (E_3_). These indicators exhibit both low impact and low levels of development. In the short term, they may be deprioritized, with resources instead allocated to urgent areas requiring improvement or refinement. Optimization of these indicators can be pursued as conditions mature.
4) Quadrant IV (Key Improvement Zone): This quadrant contains one primary indicator, the analysis layer (D), and three secondary indicators: sensing capability (A_2_), data timeliness (B_4_), and autonomous learning capability (D_5_). These indicators have significant impact on the construction sector’s digital twin technology but display low levels of development. Immediate improvements are therefore essential. Managers should allocate substantial resources—human, material, and financial—to transform these weaknesses into strengths.

Optimization Strategies for Indicators Based on Current Conditions:

1) Overall Strategic Framework

Project prioritization follows the sequence: Quadrant IV > Quadrant I > Quadrant II > Quadrant III. The investment strategy allocates over 60% of the incremental budget to areas characterized by high strategic importance but low current performance. In high-performance zones, technological leadership should be maintained; in stable zones, cost-effective optimization is recommended; and in low-priority zones, data accumulation and controlled experimentation should be sustained. An evaluation mechanism is proposed whereby all quadrant indicators undergo quarterly reassessment using the Importance–Performance Analysis (IPA) framework. This enables dynamic strategic realignment, mitigating the risk of performance degradation in high-performing areas and prolonged stagnation in underperforming zones.

2) Specific Recommendations

Indicators in the Advantage Retention Zone: It is advisable to establish a dynamic KPI monitoring dashboard that automatically aggregates key performance metrics—such as latency, accuracy, and visualization refresh rates—on a monthly basis to prevent performance volatility. Redundant computational nodes and hot backup mechanisms should be integrated into critical modules (e.g., real-time visualization and quality inspection) to ensure zero-interruption in the event of node failure. Drawing on best practices from advanced digital twin platforms in smart construction and intelligent manufacturing, the visualization rendering engine should be upgraded to support millimeter-level 4D scene reconstruction. Additionally, root cause analysis (RCA) capabilities should be embedded within production and quality management modules, enabling quality alerts to be directly linked to material batches, construction teams, and suppliers, thereby facilitating closed-loop management.

Indicators in the Steady Development Zone: Without incurring substantial investment costs, continuous optimization of network and storage performance is recommended. For instance, implementing edge caching combined with incremental data update mechanisms can alleviate transmission pressure. Cross-module standards for model and data encoding should be formulated and enforced to enhance model integration and reusability, laying the groundwork for future cross-project data fusion. Under D1/D2 indicators, scenario simulation and solution optimization algorithms should be introduced to support multi-scheme comparisons and risk assessments in resource scheduling and project timeline forecasting. Regular (quarterly or semi-annual) emergency drills for information and site safety should be conducted, with digital twin simulation results used to refine standard operating procedures (SOPs) for emergency response.

Indicators in the Low Priority Development Zone: Although short-term maintenance may be minimal, medium- to long-term breakthroughs should be strategically planned. Historical data on construction, materials, and equipment can be leveraged to deploy machine learning models that enhance early detection of risks related to progress, quality, and cost. Higher-resolution sensor data should be used to drive dynamic updates of BIM models, reducing the update cycle from biweekly to weekly or even daily. A hybrid positioning system combining Radio Frequency Identification (RFID) and Ultra-Wideband (UWB) technologies, integrated with equipment health scoring systems, can facilitate predictive maintenance and material shortage alerts. Transitioning from static views to interactive 3D/4D scenes will enable users to annotate issues and retrieve historical data directly through the visualization interface.

Indicators in the Key Improvement Zone: To enhance the detection of emergent risks, a multi-dimensional environmental sensing matrix should be established through the deployment of diverse sensors (e.g., temperature, humidity, vibration, tilt, and gas concentration). Edge computing nodes should be introduced in high-risk operational zones to enable on-site preliminary data analysis and early warning, reducing end-to-end alert latency to under 10 seconds. Online learning and transfer learning frameworks should be incorporated to allow the digital twin platform to adapt its parameters and predictive models in response to varying construction phases and environmental conditions. A closed-loop system integrating field feedback, historical deviation analysis, and model optimization should be developed to continuously improve analytical accuracy and the practical relevance of recommendations.

## 4. Discussion

### 4.1 Evaluation of the reasonableness of the results

This study analyzed the digital twin maturity of the CAZ Innovation Industrial Park project in Xinyang through an actual case study, yielding evaluation results that demonstrate the system’s strong suitability.

(1) Indicator System: The study revealed that among the primary indicators, the “Analysis Layer” accounted for the highest weight, while the “Collection Layer” carried the lowest. In terms of secondary indicators, “Self-Learning Capability” exhibited the highest global weight, whereas “Data Security Capability” ranked the lowest. These findings are consistent with the conclusions of Tao Fei et al. (2018) [99], who analyzed the hierarchical structure of digital twins and emphasized the importance and central role of the analysis layer. In contrast, the collection layer was identified as serving a more foundational function within the overall system [99].
(2) Evaluation Method: The final evaluation result of the digital twin maturity for this project, calculated using the improved matter-element model, was determined to be at the “Connected Level” (Level II). Among the primary indicators, the “Data Layer” received the highest rating, while the “Analysis Layer” scored the lowest. For the secondary indicators, “Personnel Management Capability” ranked the highest, whereas indicators such as “Model Accuracy” and “Material Management Capability” ranked the lowest. These findings align with the conclusions drawn by S. Boschert et al. [96], which emphasize the significance of data in the overall architecture of digital twins. Additionally, the analysis layer relies heavily on accumulated data and model predictions, and is therefore considered to be a relatively lower-tier element in certain cases of digital twin maturity evaluation.

Furthermore, compared to traditional models, the optimized matter-element model exhibits the following advantages:

1) Improved interpretability of boundary indicators.

As shown in Table 11, the maturity score of E6 is 90.1. While the traditional model defines a Level V classical domain as [90, 100), the optimized model, with smooth transition bands using dynamic thresholds, expands the Level I classical domain to [83, 100). Although both models yield Level I results, the correlation degree in the traditional model is merely 0.01 (which is susceptible to noise interference and abrupt grade transitions). In contrast, the optimized model achieves a correlation degree of 0.96 (indicating a 95-fold improvement in stability). This demonstrates that the optimized model not only provides greater precision in boundary responses but also significantly enhances boundary sensitivity.

Of note, dynamic thresholds also mitigate over-limit misjudgments, as mathematically proven below:

(i) Assuming the true range is [0, 100], the extended Level V domain is [83,100].
(ii) For over-limit values 𝜇∈ [90,100], the proportion of the actual effective range is:

$$\frac{\text{1}\text{0}\text{0}-\text{9}\text{0}}{\text{1}\text{0}\text{0}-\text{8}\text{3}}\times \text{1}\text{0}\text{0}\%=\text{5}\text{8}.\text{8}\%$$

This indicates that over-limit values retain a reasonable affiliation within the extended domain, avoiding forced classification into the highest level.

2) Enhanced Clarity in Correlation Degree Interpretation.

The optimized model achieves greater clarity in the representation of correlation degrees. By mapping the original correlation values—including potentially negative values—onto the [0,1] interval, the model effectively eliminates the distortions introduced by negative correlation degrees in traditional methods. This nonlinear mapping not only improves the intuitive interpretability of the results but also aligns the correlation measures more closely with probabilistic semantics, thereby enhancing their comprehensibility and practical applicability.

For example, as presented in Tables 11 and 13, the maturity score of indicator C1 is 46.5. Under the traditional model, the corresponding correlation degree is −0.15 (Level V), which could easily be misinterpreted as indicating a “strong negative correlation.” In contrast, within the optimized model, this value is remapped to 0.852 (Level V), thereby providing a more accurate reflection of its actual influence (refer to Table 15). Such a transformation substantially improves both the continuity and the rationality of the evaluation results, effectively preventing misclassification stemming from inappropriate sign conventions or scale definitions.

**Table 15:** Optimized correlation degree matrix for secondary indicators.

Code	Correlation Degree at Level I	Correlation Degree at Level II	Correlation Degree at Level III	Correlation Degree at Level IV	Correlation Degree at Level V
A_1_	0.412	0.682	0.912	0.732	0.521
A_2_	0.428	0.892	0.723	0.615	0.402
A_3_	0.398	0.532	0.752	0.908	0.563
B_1_	0.41	0.675	0.911	0.728	0.518
B_2_	0.385	0.935	0.642	0.482	0.712
B_3_	0.387	0.487	0.648	0.932	0.708
B_4_	0.712	0.925	0.351	0.285	0.398
B_5_	0.422	0.718	0.881	0.602	0.412
B_6_	0.698	0.932	0.342	0.265	0.395
C_1_	0.852	0.102	0.018	0.035	0.041
C_2_	0.408	0.655	0.905	0.741	0.538
C_3_	0.685	0.942	0.328	0.248	0.392
C_4_	0.425	0.715	0.883	0.598	0.408
C_5_	0.43	0.709	0.891	0.592	0.405
D_1_	0.435	0.702	0.902	0.585	0.398
D_2_	0.43	0.709	0.891	0.592	0.405
D_3_	0.445	0.925	0.682	0.558	0.388
D_4_	0.425	0.715	0.883	0.598	0.408
D_5_	0.45	0.912	0.665	0.542	0.382
E_1_	0.409	0.662	0.904	0.734	0.526
E_2_	0.425	0.715	0.883	0.598	0.408
E_3_	0.838	0.118	0.026	0.038	0.043
E_4_	0.411	0.672	0.903	0.726	0.522
E_5_	0.395	0.515	0.724	0.928	0.702
E_6_	0.382	0.465	0.618	0.715	0.962
E_7_	0.384	0.475	0.635	0.942	0.728

(3) Feedback Mechanism: The Importance-Performance Analysis (IPA) identified the “Analysis Layer” as the key area of focus, suggesting priority improvements in sensor capability, data real-time performance, and self-learning capability indicators. These findings are consistent with the conclusions of W. Kritzinger et al. [100], who explicitly emphasized that sensor technology and data real-time performance are critical elements for enhancing the accuracy of digital twins. Furthermore, the integration of self-learning algorithms allows digital twins to achieve more efficient data processing and intelligent decision-making.

This project employed the Importance–Performance Analysis (IPA) framework to optimize resource allocation, thereby facilitating the efficient implementation of a digital twin platform in the context of construction engineering. As demonstrated in Table 16, the strategy of prioritizing breakthroughs in critical improvement zones, consolidating performance in high-value areas, implementing cost-effective enhancements in stable zones, and maintaining strategic oversight in low-priority zones yielded outcomes that significantly exceeded initial expectations. Specifically, construction efficiency improved by 30%, the project timeline was shortened by nearly seven months, and cumulative cost savings surpassed RMB 16 million. These results provide robust empirical validation of the feasibility and forward-looking nature of IPA-guided strategies in enhancing both the economic performance and technological maturity of construction projects. The findings offer a replicable model and decision-making reference for future smart construction site development.

**Table 16:** Projected benefits vs. actual efficiency gains.

Category	Projected Benefit	Actual Efficiency Gain
Time Objective	24 months	17 months
Equipment Procurement and Rental Costs	RMB 4.00 million saved	RMB 5.18 million saved
Construction Materials and Prefabricated Parts	RMB 3.00 million saved	RMB 3.63 million saved
Labor Costs	RMB 1.00 million saved	RMB 1.56 million saved
Economic Output from Labor, Materials, Equipment	RMB 2.00 million increase	RMB 3.09 million increase
Overall Construction Efficiency (vs. traditional)	25% improvement	30% improvement
Schedule and Management Costs	RMB 2.00 million saved	RMB 3.06 million saved

### 4.2 Expansion of the appropriateness of the research framework

The proposed research framework demonstrates robust generalizability and adaptability across diverse regulatory and technological environments, contingent upon localized adjustments that preserve its structural integrity and evaluative rigor.

(1) In regulatory contexts marked by legal heterogeneity, the framework must reconcile compliance requirements with engineering feasibility. The European Union’s General Data Protection Regulation (GDPR) imposes strict mandates on data quality, storage security, and data subject rights—including data minimization, anonymization, and breach notification within 72 hours. To operationalize these mandates, indicators such as anonymization coverage, degree of data minimization, and response timeliness should be embedded within the data layer, with dynamically adjusted weights to support privacy-by-design configurations [101,102]. A cost–benefit calibration mechanism is also essential to quantify the social welfare gains and implementation costs of privacy protection, thereby mapping these metrics to indicator thresholds and weights to avoid overregulation that may suppress data utility [103]. In contrast, the United States’ decentralized, sector-specific, and technology-driven governance model necessitates modular compliance and interoperability assessments. These should focus on interface standardization, semantic consistency, security validation, and cross-platform coordination, aligning with research trajectories in AEC semantic interoperability and semantic digital twins [20,88]. In emerging markets such as China, where policy emphasizes technological deployment and industrialization, the framework must prioritize technical integration and capacity building. Data and network security, along with industry-specific compliance, should be deeply embedded in indicator design. In high-density digital twin scenarios, joint constraints involving data, computational power, and connectivity must be holistically considered [86,94].
(2) Given the dependency of digital twin maturity on data infrastructure and computational ecosystems, a stratified adaptation strategy is required. In low-resource environments, manual data entry may be permitted with credibility discounts, and thresholds for model accuracy and analytical latency may be relaxed. Intermediate conditions, often constrained by system silos and limited predictive capabilities, demand emphasis on connection efficiency, interface automation, and data backfill. High-resource environments should prioritize predictive control, real-time feedback, and closed-loop decision-making, supported by contingency protocols and manual review to mitigate risks associated with over-technologization [86,94]. Semantic interoperability should serve as the foundational layer, with ontology and semantic web technologies ensuring cross-platform consistency in data and model semantics [20,88]. Indicator design should draw from tiered maturity frameworks to structurally configure weights and thresholds, aligning the five-layer capability distribution with the project’s technological stage [87].
(3) Project scale and contractual models further shape the depth and focus of evaluation. Small-scale projects typically emphasize core data acquisition and basic visualization, allowing for simplified indicator sets. Large and complex projects require assessment of advanced analytics and comprehensive data integration, consistent with empirical findings from digital twin capability stratification and BIM maturity frameworks [87]. Under Design–Bid–Build (DBB) models, characterized by elongated information chains and complex cross-organizational coordination, indicators should reinforce seamless design–construction integration, model conversion accuracy, and collaborative update mechanisms. The data layer should emphasize standard compliance, timeliness, and consistency in inter-organizational exchange, while the application layer should address digital change management and multi-party decision-making maturity. IPA analyses frequently identify the modeling and data layers as critical improvement domains [89,93,104]. Integrated delivery models such as DB/EPC and IPD, which feature centralized decision chains and collaborative emphasis, require elevated weighting of the analysis layer, focusing on real-time cost–schedule–quality analytics and predictive risk alerts. The application layer should support centralized command and rapid closure, while the modeling layer must enable collaborative design and model-driven construction. Comparative studies have demonstrated the efficiency and performance advantages of integrated delivery, providing empirical justification for these configurations [92]. Relational contracts and joint risk management mechanisms can further enhance incentive compatibility by linking data sharing and predictive control to production management and risk alert indicators [105].
(4) To address cultural and organizational barriers in global deployment, a multi-dimensional strategy is essential. Trust and data governance should be reinforced through cross-organizational data sharing agreements, stratified responsibilities, and auditable catalogs, clarifying ownership, usage boundaries, and value distribution. Privacy-enhancing technologies such as anonymization and differential privacy should enable risk-tiered openness, with performance-linked incentives incorporating data contributions [90,106]. Organizational transformation and capability gaps must be addressed via tiered maturity frameworks that define staged competencies across acquisition, data, modeling, analysis, and application layers. Scenario-based training and change management initiatives can improve BIM/DT adoption and collaborative performance [91]. Semantic consistency should be ensured through domain ontologies and minimal viable semantic sets, with cross-platform fidelity and interoperability regression testing incorporated into evaluation metrics [20,88]. Privacy and ethical considerations require embedding indicators such as anonymization coverage, data minimization, response latency, and explainable appeal mechanisms within the data layer, with dynamic weighting based on jurisdictional context [101,102]. Economic trade-offs must be considered to prevent excessive constraints that undermine data value. Incentive misalignment and risk preferences should be addressed through relational contracts and joint risk management, binding predictive analytics and data sharing to compensation, risk allocation, and performance incentives. Indicator weights should be adjusted according to delivery models to reflect decision chain characteristics [90,105]. Finally, thresholds and weights must be contextually configured based on technological infrastructure. In low-to-medium resource settings, priority should be given to connectivity and data quality enhancement; in high-resource environments, emphasis should be placed on predictive control and real-time closure. IPA should be employed to identify high-importance, low-performance gaps, guiding iterative optimization [86,94].

### 4.3 Technical challenges and mitigation strategies

While the proposed evaluation framework leverages real-time data as a core advantage, its large-scale deployment may encounter several technical challenges. To ensure system stability, scalability, and assessment accuracy, this section systematically identifies potential issues across five critical technical dimensions and proposes targeted mitigation strategies.

(1) Sensor Network Bandwidth Limitations

High-frequency, multi-source sensor data uploads can lead to link congestion and packet loss, constraining sampling rates and node scalability while amplifying end-to-end transmission bottlenecks [107]. To mitigate these issues, four strategies are proposed: (i) edge computing should be deployed near the data source to perform denoising, feature extraction, and lightweight inference, thereby reducing upstream traffic [108]; (ii) data compression and adaptive encoding techniques—such as keyframe extraction, point cloud voxelization, and differential compression—can minimize bandwidth consumption within acceptable error margins [109]; (iii) selective acquisition and in-network aggregation, enabled by event-triggered sampling and node-side filtering, ensure that only informative data variations are transmitted [110]; and (iv) event-driven control mechanisms can replace periodic full reporting, actively reducing link load [111]. These strategies collectively establish an efficient and controllable data acquisition and transmission architecture that enhances system responsiveness and reliability.

(2) Data Latency and Jitter

Multi-hop routing and protocol stack overhead may impair real-time feedback and online collaborative simulation, making it difficult to maintain control and alert loops within millisecond-to-second latency budgets [112]. To address this, four optimization strategies are recommended: (i) edge–cloud layered closed-loop control, where high-frequency control logic is executed at the edge while the cloud handles low-frequency global optimization [108]; (ii) end-to-end QoS and flow control mechanisms, incorporating service-level differentiation and congestion feedback, to suppress queuing delays [113]; (iii) prioritized transmission of keyframes and event-driven data to ensure timely reporting during anomalies [111]; and (iv) high-precision time synchronization across sources, achieved through clock alignment and temporal calibration, to alleviate processing delays caused by time drift [114]. These measures collectively enhance system responsiveness and support stable collaborative simulation.

(3) Computational Resource Constraints

Real-time stream processing, complex event detection, 3D simulation, and machine learning inference impose intensive demands on GPU, memory, and I/O resources. Centralized deployment may lead to performance bottlenecks and resource contention [115]. To alleviate this, four resource optimization strategies are proposed: (i) edge intelligence and distributed model deployment to offload computationally intensive tasks to peripheral nodes [108]; (ii) hybrid simulation strategies using high-fidelity models for critical scenarios and reduced-order or surrogate models elsewhere to balance accuracy and efficiency [116]; (iii) feature-level data uploads, rather than raw streams, to reduce computational and I/O burdens [109]; and (iv) elastic stream processing architectures incorporating load scaling and window-based scheduling to smooth peak system loads [113]. These strategies contribute to a scalable and efficient computational framework capable of supporting real-time evaluation and simulation in complex environments.

(4) Data Fusion Complexity

Integrating multi-source heterogeneous data presents significant challenges in synchronization, coordinate transformation, semantic alignment, and precision control, which affect the consistency and predictive reliability of the digital twin [111]. To address this, three fusion optimization strategies are proposed: (i) robust fusion mechanisms based on uncertainty modeling—using confidence scores and covariance matrices—to manage conflicts and enhance data consistency [117]; (ii) high-precision clock synchronization and spatiotemporal alignment techniques, such as PTP-level timing and sliding window calibration, to mitigate cross-source drift [114]; and (iii) unified semantic and data standards, including domain-specific ontologies for the built environment, to reduce integration friction and ensure semantic coherence [118]. These approaches significantly improve the accuracy and consistency of data fusion, thereby supporting high-quality modeling and prediction.

(5) Architectural and Engineering Support Deficiencies

The absence of end-to-end QoS guarantees, flow control mechanisms, standardized metadata, and tiered data management strategies may lead to cascading degradation and traceability loss during scalable deployment [110,111]. To address this, three systemic improvement strategies are proposed: (i) integrated QoS and backpressure mechanisms enabling priority-based scheduling and congestion-aware traffic management, with edge-side aggregation to reduce network load [110,113]; (ii) event-triggered governance mechanisms to replace periodic full reporting and prevent systemic congestion [111]; and (iii) a standardized governance framework—including unified metadata structures, semantic standards, and data lineage tracking—to enhance system traceability and interoperability [20]. These improvements establish a stable and scalable architecture capable of supporting reliable operation in complex engineering environments.

### 4.4 Innovations and limitations of the study

(1) Innovations: Existing research on the evaluation of digital twin maturity during the construction phase remains limited in terms of objective and quantitative evaluation methods and standards, with relatively few practical cases available. This study makes significant contributions to the methodology and empirical case analysis within the domain of digital twin maturity evaluation in construction. The innovations of this study are summarized as follows:
  1) This research provides a universal methodological framework to support the evaluation of digital twin maturity in the global construction sector. The proposed comprehensive evaluation process facilitates the hierarchical processing of complex maturity assessment systems, offering simplicity in calculation, relative objectivity, and practical applicability. It enhances the accuracy of the maturity evaluation system during the construction phase and lays a solid foundation for further research on construction-phase maturity assessment.
  2) The case study presented in this research fills a gap in empirical studies on digital twin maturity evaluation within the construction field. It offers practical insights for researchers and professionals in this area, helping to advance the development of digital twins in the global construction industry and providing reliable references for similar projects.
(2) Limitations: This study primarily focuses on the application of digital twin technology during the construction phase, with an emphasis on progress and quality management. However, it does not incorporate the monitoring or analysis of material performance, thereby limiting the ability to directly correlate macro-level construction outcomes with micro-level material mechanisms. Prior research has demonstrated that the incorporation of polyaluminum chloride waste residue and citric acid into magnesium oxychloride cement can significantly enhance its water resistance, volumetric stability, and pore structure, while also delaying the setting time [118]. These findings suggest that future digital twin frameworks should integrate key material performance parameters to improve the comprehensiveness and explanatory power of the evaluation system. Moreover, the absence of a standardized evaluation framework for digital twin applications in China presents an additional challenge. Future research should aim to synthesize the latest global policy documents related to digital twin technologies and expand the evaluation scope to encompass the entire building lifecycle. Through extensive case-based analysis, the effectiveness of digital twin implementation can be systematically assessed. Consequently, the maturity assessment indicators should be dynamically updated in accordance with the evolving characteristics and specific objectives of digital twin technologies.

## 5. Conclusion

This study developed a novel quantifiable evaluation model for digital twin maturity, primarily aimed at assessing the maturity level of digital twins during the construction phase. The main conclusions are as follows:

(1) Evaluation System: Based on the characteristics of digital twins during the construction phase, this study proposed a maturity model encompassing five dimensions—Collection Layer, Data Layer, Modeling Layer, Analysis Layer, and Application Layer—and 26 evaluation indicators. Five levels of maturity were defined as the classification criteria. During the weighting process, the AHP-Entropy method was utilized to effectively overcome the subjectivity of data. In the comprehensive evaluation phase, an optimized matter-element model based on dynamic thresholds and nonlinear correlation degrees was introduced to resolve incompatibilities in complex systems more effectively and improve evaluation accuracy. In the feedback phase, the IPA method was employed for evaluation result analysis, enabling more intuitive optimization of the outcomes.
(2) Case Study: Through an analysis of the CAZ Innovation Industrial Park project in Xinyang, Henan Province, the digital twin maturity was determined to be at the Connected Level (Level II). Primary indicators require prioritized attention to the “Analysis Layer,” while secondary indicators call for improvements in sensor capability, data real-time performance, and self-learning capability within digital twin systems. Empirical validation demonstrated that the proposed maturity evaluation model is highly operable, providing accurate assessment results and optimization recommendations for the enterprise’s digital twin level and effectively guiding the application of digital twin technology.

The digital twin maturity model proposed in this study not only advances the development and application of digital twin technology during the construction phase but also provides both theoretical and practical value for the progress of digital twin technology within the construction domain. This model serves as a reference for the implementation of similar projects and offers the academic community a novel approach for further research in this field.

## Supporting information

S1 Appendix Determination of δ_*j*_. (DOCX)

S2 Appendix Sensitivity Analysis. (DOCX)

S3 File Raw and processed data files. (XLSX)
