# Supplementary material for: Constructing a digital twin maturity assessment framework for the building construction phase based on an improved matter-element model: A case study of a construction project in Xinyang, China
Source: PLoS One. 2025 Sep 29;20(9):e0332449. doi: 10.1371/journal.pone.0332449 (PMC12478936; doi:10.1371/journal.pone.0332449)
Supplement: S1 Appendix — (DOCX) [file pone.0332449.s001.docx]

S1 Appendix： Determination of 𝛿𝑗

According to the JCGM 100:2008 Guide to the Expression of Uncertainty in Measurement (GUM), when a uniform distribution is assumed, the standard uncertainty is given by

$$u=\frac{a}{\sqrt{3}}$$

where 𝑎 is the half-width of the interval[A1].

Referring to ISO/IEC Guide 98-3:2008 – Uncertainty of Measurement, Part 3 (https://www.iso.org/standard/50461.html ), in industrial applications the permissible error range is typically ±5 % of the measurement span. This corresponds to a total width of 10 %, hence 𝑎=0.1|𝑋𝑗|. Therefore:

$$u=\frac{0.1\left| X_{j} \right|}{\sqrt{3}}\approx0.05\left| X_{j} \right|$$

Following the National Institute of Standards and Technology (NIST) guidelines (NIST Technical Note 1900) (<https://nvlpubs.nist.gov/nistpubs/technicalnotes/nist.tn.1900.pdf> ), a coverage factor 𝑘=2 should be applied to expand the standard uncertainty 𝑢 to the expanded uncertainty 𝑈, providing approximately a 95 % confidence level:

$$U=k\times u\approx0.1\left| X_{j} \right|$$

According to the International Society of Automation (ISA) recommended practice ISA‑RP74.02: Methodology for the Determination of Uncertainty in Industrial Process Measurements (<https://www.isa.org/>),

𝛿_𝑗_=(0.5 to 1.0)×𝑈

In this study, we adopt

𝛿𝑗= (0.05 to 0.1)×|𝑋𝑗|

which corresponds to 50 %–100 % of the expanded uncertainty, thereby covering most of the uncertainty while avoiding excessive expansion.
