# Supplementary material for: Constructing a digital twin maturity assessment framework for the building construction phase based on an improved matter-element model: A case study of a construction project in Xinyang, China
Source: PLoS One. 2025 Sep 29;20(9):e0332449. doi: 10.1371/journal.pone.0332449 (PMC12478936; doi:10.1371/journal.pone.0332449)
Supplement: S2 Appendix — (DOCX) [file pone.0332449.s002.docx]

**S2 Appendix：Sensitivity Analysis**

The parameter 𝛿_𝑗_ was set as 𝑐×|𝑋_𝑗_|, with 𝑐 values of 0.05, 0.06, 0.07, 0.08, 0.09, and 0.10 tested for validation.

(i) Determination of the classical domain

The classical domains obtained under different 𝛿_𝑗_ values are shown in Table A.

**Table A. Classical Domains Corresponding to the Six 𝛿_𝑗_ Values.**

| Level | *c*=0.05 | *c*=0.06 | *c*=0.07 | *c*=0.08 | *c*=0.09 | *c*=0.10 |
| --- | --- | --- | --- | --- | --- | --- |
| I | [0, 65) | [0, 66) | [0, 67) | [0, 68) | [0, 69) | [0, 70) |
| II | [55, 75) | [54, 76) | [53, 77) | [52, 78) | [51, 79) | [50, 80) |
| III | [65, 85) | [64, 86) | [63, 87) | [62, 88) | [61, 89) | [60, 90) |
| IV | [75, 95) | [74, 96) | [73, 97) | [72, 98) | [71, 99) | [70, 100) |
| V | [85, 100] | [84, 100] | [83, 100] | [82, 100] | [81, 100] | [80, 100] |

(ii) Response of Indicators

As shown in Table B, when 𝛿𝑗=0.5|𝑋_𝑗_|, 𝛿𝑗=0.6|𝑋_𝑗_|, 𝛿𝑗=0.7|𝑋_𝑗_|, and 𝛿𝑗=0.8|𝑋_𝑗_|, the misclassification rate of indicator levels remains at 0%, indicating the rationality of all four threshold settings. Notably, under the condition 𝛿𝑗=0.7|𝑋_𝑗_|, 84.62% of indicator levels correspond to the maximum correlation degree values, suggesting that this threshold yields the clearest level differentiation.

**Table B. Maturity Level Responses of Indicators Under Different 𝛿_𝑗_ Thresholds.**

| Code | Expert Rating Level | Score | *c*=0.05 | *c*=0.06 | *c*=0.07 | *c*=0.08 | *c*=0.09 | *c*=0.10 |
| --- | --- | --- | --- | --- | --- | --- | --- | --- |
| A_1_ | III | 76.5 | 0.82 (III) | 0.86 (III) | 0.91 (III) | 0.87 (III) | 0.84 (III) | 0.81 (III) |
| A_2_ | II | 68.7 | 0.68 (II) | 0.75 (II) | 0.89 (II) | 0.80 (II) | 0.74 (II) | 0.63 (II) |
| A_3_ | IV | 80.1 | 0.75 (IV) | 0.84 (IV) | 0.90 (IV) | 0.79 (IV) | 0.70 (V) | 0.62 (V) |
| B_1_ | III | 75.8 | 0.79 (III) | 0.87(III) | 0.91(III) | 0.89(III) | 0.83(III) | 0.77(III) |
| B_2_ | IV | 85.1 | 0.91 (IV) | 0.92 (IV) | 0.94 (IV) | 0.94 (IV) | 0.91 (IV) | 0.88 (IV) |
| B_3_ | IV | 84.4 | 0.88 (IV) | 0.90 (IV) | 0.93 (IV) | 0.92 (IV) | 0.89 (IV) | 0.84 (IV) |
| B_4_ | II | 64.4 | 0.62 (II) | 0.79 (II) | 0.92 (II) | 0.91 (II) | 0.84 (II) | 0.65 (II) |
| B_5_ | III | 73.7 | 0.71 (III) | 0.81 (III) | 0.88 (III) | 0.82(III) | 0.79 (III) | 0.72 (III) |
| B_6_ | II | 63 | 0.55 (II) | 0.69 (II) | 0.93 (II) | 0.88 (II) | 0.76 (II) | 0.65 (II) |
| C_1_ | I | 46.5 | 0.58 (I) | 0.75 (I) | 0.85 (I) | 0.77 (I) | 0.71 (I) | 0.62 (I) |
| C_2_ | III | 78.7 | 0.93 (III) | 0.91 (III) | 0.91 (III) | 0.88 (III) | 0.82 (III) | 0.79 (III) |
| C_3_ | II | 61.5 | 0.60 (II) | 0.85 (II) | 0.94 (II) | 0.89 (II) | 0.78 (II) | 0.64 (II) |
| C_4_ | III | 73 | 0.75 (III) | 0.81 (III) | 0.88 (III) | 0.88 (III) | 0.80 (III) | 0.73 (III) |
| C_5_ | III | 71.5 | 0.68 (III) | 0.78 (III) | 0.89 (III) | 0.86 (III) | 0.77 (III) | 0.67 (III) |
| D_1_ | III | 70.1 | 0.65 (III) | 0.80 (III) | 0.90 (III) | 0.90 (III) | 0.82 (III) | 0.70 (III) |
| D_2_ | III | 71.5 | 0.73 (III) | 0.82 (III) | 0.89 (III) | 0.87 (III) | 0.81 (III) | 0.74 (III) |
| D_3_ | II | 67.2 | 0.57 (II) | 0.69 (II) | 0.93 (II) | 0.92 (II) | 0.84 (II) | 0.62 (II) |
| D_4_ | III | 73 | 0.75 (III) | 0.83 (III) | 0.88 (III) | 0.84 (III) | 0.74 (III) | 0.68 (III) |
| D_5_ | II | 65.8 | 0.52 (II) | 0.74 (II) | 0.91 (II) | 0.92 (II) | 0.82 (II) | 0.60 (II) |
| E_1_ | III | 78 | 0.95 (III) | 0.90 (III) | 0.90 (III) | 0.86 (III) | 0.82 (III) | 0.79 (III) |
| E_2_ | III | 73 | 0.75 (III) | 0.88 (III) | 0.88 (III) | 0.84 (III) | 0.76 (III) | 0.70 (III) |
| E_3_ | I | 50.1 | 0.63 (I) | 0.74 (I) | 0.84 (I) | 0.84 (I) | 0.75 (I) | 0.56 (II) |
| E_4_ | III | 77.2 | 0.92 (III) | 0.90 (III) | 0.90 (III) | 0.87 (III) | 0.86 (III) | 0.80 (III) |
| E_5_ | IV | 80.8 | 0.82 (IV) | 0.89 (IV) | 0.93 (IV) | 0.93 (IV) | 0.87 (IV) | 0.73 (IV) |
| E_6_ | V | 90.1 | 0.78 (V) | 0.94 (V) | 0.96 (V) | 0.90 (V) | 0.86 (V) | 0.81 (V) |
| E_7_ | IV | 86.5 | 0.89 (IV) | 0.94 (IV) | 0.94 (IV) | 0.94 (IV) | 0.91 (IV) | 0.84 (IV) |
| False Positive Rate/% | | | 0 | 0 | 0 | 0 | 3.85 | 7.69 |
| Proportion of Maximum Correlation Values (Including Identical Values) / % | | | 11.54 | 7.69 | 84.62 | 23.08 | 0 | 0 |

（iii）Sensitivity of Indicators

Sensitivity reflects the extent to which the model output (i.e., correlation degree) responds to variations in the input (i.e., indicator scores). The expression is as follows:

$$S_{j}=\left| \frac{\vartheta K}{\vartheta x_{j}} \right|_{x=a_{k}}$$

•$S_{j}$—Sensitivity of the 𝑗-th indicator

•*K* — Model output (correlation degree).

•*x_j_—* The 𝑗-th input indicator.

•$\frac{\vartheta K}{\vartheta x}$— The partial derivative of 𝐾 with respect to 𝑥, describing the local rate of change of 𝐾 as 𝑥 changes infinitesimally.

• | |— Absolute value operator, ensuring that the sensitivity is expressed as a magnitude without regard to the sign of change.

*• a_k_* — The 𝑘-th boundary point.

As shown in Table C, sensitivity decreases monotonically with increasing values of 𝛿_𝑗._ Specifically, smaller values of 𝛿_𝑗_ (e.g., 0.05|𝑋_𝑗_| or 0.06|𝑋_𝑗_|) meet the requirements for high sensitivity and are suitable for scenarios that demand the detection of subtle variations. In contrast, larger values of 𝛿_𝑗_ (e.g., 0.09|𝑋_𝑗_|or 0.10|𝑋_𝑗_|) prioritize system stability, making them appropriate for noise-prone environments by reducing false alarm rates and enhancing robustness. Intermediate values of 𝛿𝑗 (e.g., 0.07|𝑋𝑗| or 0.08|𝑋𝑗|) offer a balanced trade-off between sensitivity and stability, and are thus well-suited for most practical applications.

**Table C.** **Sensitivity Variation Trends of Each Indicator under Different Values of 𝛿_𝑗。_**

| Code | c=0.05 | c=0.06 | c=0.07 | c=0.08 | c=0.09 | c=0.10 |
| --- | --- | --- | --- | --- | --- | --- |
| A1 | 0.0421 | 0.0413 | 0.0406 | 0.0399 | 0.0392 | 0.0386 |
| A2 | 0.0387 | 0.0381 | 0.0375 | 0.0369 | 0.0363 | 0.0357 |
| A3 | 0.0453 | 0.0445 | 0.0437 | 0.043 | 0.0423 | 0.0416 |
| B1 | 0.0418 | 0.041 | 0.0403 | 0.0396 | 0.0389 | 0.0383 |
| B2 | 0.0462 | 0.0454 | 0.0446 | 0.0438 | 0.0431 | 0.0424 |
| B3 | 0.0458 | 0.045 | 0.0442 | 0.0434 | 0.0427 | 0.042 |
| B4 | 0.0372 | 0.0366 | 0.036 | 0.0354 | 0.0348 | 0.0342 |
| B5 | 0.0405 | 0.0398 | 0.0391 | 0.0384 | 0.0377 | 0.0371 |
| B6 | 0.0368 | 0.0362 | 0.0356 | 0.035 | 0.0344 | 0.0338 |
| C1 | 0.0321 | 0.0315 | 0.031 | 0.0305 | 0.03 | 0.0295 |
| C2 | 0.0432 | 0.0424 | 0.0416 | 0.0409 | 0.0402 | 0.0396 |
| C3 | 0.0365 | 0.0359 | 0.0353 | 0.0347 | 0.0341 | 0.0335 |
| C4 | 0.0402 | 0.0395 | 0.0388 | 0.0381 | 0.0374 | 0.0367 |
| C5 | 0.0398 | 0.0391 | 0.0384 | 0.0377 | 0.037 | 0.0363 |
| D1 | 0.0389 | 0.0382 | 0.0375 | 0.0369 | 0.0363 | 0.0357 |
| D2 | 0.0398 | 0.0391 | 0.0384 | 0.0377 | 0.037 | 0.0363 |
| D3 | 0.0381 | 0.0375 | 0.0369 | 0.0363 | 0.0357 | 0.0351 |
| D4 | 0.0402 | 0.0395 | 0.0388 | 0.0381 | 0.0374 | 0.0367 |
| D5 | 0.0378 | 0.0372 | 0.0366 | 0.036 | 0.0354 | 0.0348 |
| E1 | 0.043 | 0.0422 | 0.0414 | 0.0407 | 0.04 | 0.0394 |
| E2 | 0.0402 | 0.0395 | 0.0388 | 0.0381 | 0.0374 | 0.0367 |
| E3 | 0.0328 | 0.0322 | 0.0316 | 0.0311 | 0.0306 | 0.0301 |
| E4 | 0.0425 | 0.0417 | 0.0409 | 0.0402 | 0.0395 | 0.0389 |
| E5 | 0.0456 | 0.0448 | 0.044 | 0.0432 | 0.0425 | 0.0418 |
| E6 | 0.0478 | 0.0469 | 0.0461 | 0.0453 | 0.0445 | 0.0438 |
| E7 | 0.0465 | 0.0456 | 0.0448 | 0.044 | 0.0432 | 0.0425 |
| Mean Sensitivity | 0.0407 | 0.04 | 0.0393 | 0.0386 | 0.038 | 0.0374 |
| Trend in Mean Sensitivity | Baseline | -1.70% | -3.40% | -5.20% | -6.60% | -8.10% |

References A

[A1] Joint Committee for Guides in Metrology. Evaluation of measurement data—Guide to the expression of uncertainty in measurement. JCGM 100:2008. Sèvres (France): BIPM; 2008.
